# The senescence-associated secretome of Hedgehog-deficient hepatocytes drives MASLD progression

**DOI:** 10.1172/JCI180310

**Published:** 2024-08-27

**Authors:** Ji Hye Jun, Kuo Du, Rajesh Kumar Dutta, Raquel Maeso-Diaz, Seh Hoon Oh, Liuyang Wang, Guannan Gao, Ana Ferreira, Jon Hill, Steven S. Pullen, Anna Mae Diehl

**Affiliations:** 1 Division of Gastroenterology, Department of Medicine and; 2 Department of Molecular Genetics and Microbiology, Duke University, Durham, North Carolina, USA.; 3 Boehringer Ingelheim Pharmaceuticals Inc., Ridgefield, Connecticut, USA.

**Keywords:** Hepatology, Metabolism, Cell stress, Cellular senescence, Mitochondria

## Abstract

The burden of senescent hepatocytes correlates with the severity of metabolic dysfunction–associated steatotic liver disease (MASLD), but the mechanisms driving senescence and how it exacerbates MASLD are poorly understood. Hepatocytes experience lipotoxicity and become senescent when Smoothened (Smo) is deleted to disrupt Hedgehog signaling. We aimed to determine whether the secretomes of Smo-deficient hepatocytes perpetuate senescence to drive MASLD progression. RNA-Seq analysis of liver samples from human and murine cohorts with MASLD confirmed that hepatocyte populations in MASLD livers were depleted of Smo^+^ cells and enriched with senescent cells. When fed a choline-deficient, amino acid–restricted high-fat diet (CDA-HFD) to induce MASLD, Smo^–^ mice had lower antioxidant markers and developed worse DNA damage, senescence, steatohepatitis, and fibrosis than did Smo^+^ mice. Sera and hepatocyte-conditioned medium from Smo^–^ mice were depleted of thymidine phosphorylase (TP), a protein that maintains mitochondrial fitness. Treating Smo^–^ hepatocytes with TP reduced senescence and lipotoxicity, whereas inhibiting TP in Smo^+^ hepatocytes had the opposite effect and exacerbated hepatocyte senescence, steatohepatitis, and fibrosis in CDA-HFD–fed mice. We conclude that inhibition of Hedgehog signaling in hepatocytes promoted MASLD by suppressing hepatocyte production of proteins that prevent lipotoxicity and senescence.

## Abstract 2

Hepatocyte hedgehog signaling generates factors that maintain metabolic homeostasis and prevent metabolic dysfunction-associated steatotic liver disease (MASLD).

## Graphical abstract

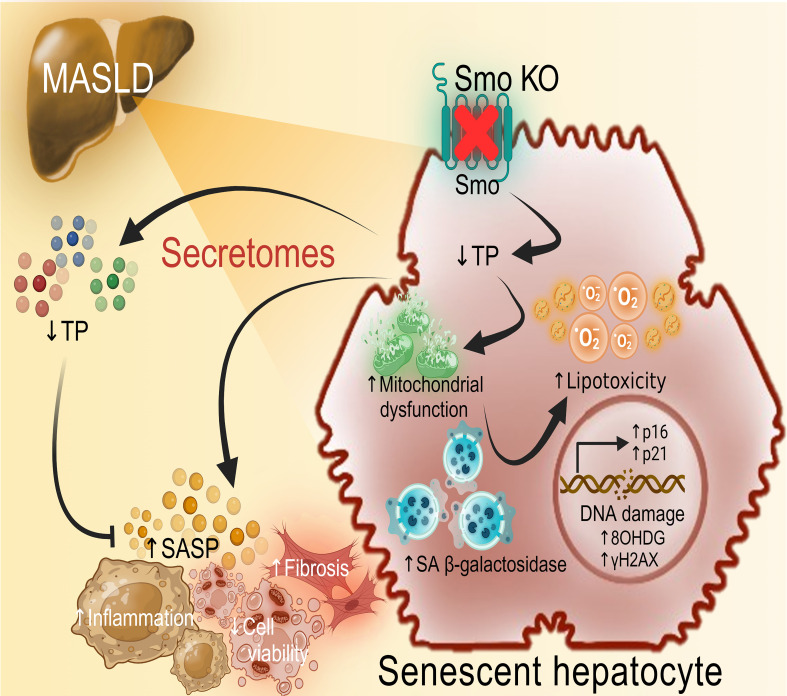

## Introduction

Over the past 50 years, much of the world’s population has transitioned into a state of relative energy surplus due to improvements in food availability and declining demands for physical activity. This shift has imposed challenges on homeostatic mechanisms that maintain tissue energy balance, spawning a global increase in diseases caused by metabolic dysfunction (1). Metabolic dysfunction–associated steatotic liver disease (MASLD) is now a leading cause of chronic liver disease in most countries (2). Liver-related mortality in MASLD is greatest in people with MASLD cirrhosis and associates with increased mortality from other causes (3). MASLD cirrhosis is the end result of a chronic degenerative process that develops when constitutive regenerative mechanisms to replace dead hepatocytes are overwhelmed, leading to progressive replacement of functional parenchyma with fibrous scarring. Thus, development of interventions to prevent, arrest, and/or reverse MASLD progression requires a deeper understanding of the inherent mechanisms that normally maintain liver homeostasis.

The risk for MASLD cirrhosis and other metabolic dysfunction–related degenerative diseases varies among individuals but generally increases with age (4). Older age increases the incidence and prevalence of degenerative diseases because epigenetic modifications that promote cellular metabolic dysfunction accumulate with aging. This amplifies the tissue burden of dysfunctional cells that are unable to regenerate but that secrete factors that perpetuate maladaptive repair. Thus, cellular senescence and related senescence-associated secretory phenotypes (SASPs) are acknowledged hallmarks of aging tissues (5). MASLD and other chronic liver diseases induce hepatocyte DNA damage and senescence, and MASLD severity correlates with the burden of senescent hepatocytes (6). However, the mechanisms driving hepatocyte senescence, and how this exacerbates MASLD, are poorly understood.

Hedgehog, a lipid-regulated morphogenic signaling pathway, is known to inhibit senescence and promote longevity (7). Hedgehog signaling is tightly coupled to primary cilia and, thus, nutrient sensing (8). Hedgehog maintains organelle quality control mechanisms and optimizes energy retrieval while repressing expression of cell-cycle inhibitors (9). Hence, growing and regenerating tissues, as well as many cancers, exhibit high Hedgehog pathway activity (10). Previous studies demonstrated increased Hedgehog signaling in chronically injured livers and liver cancers, but barely any detectable pathway activity in healthy livers, consistent with evidence that hepatocytes replicate very infrequently in adulthood (11). Also, as mentioned earlier, Hedgehog signaling is tightly coupled to cilia, and cilia are found in fewer than 10% of adult hepatocytes at a given time (12). Together, these findings led to the assumption that the Hedgehog pathway is dormant in the vast majority of healthy adult hepatocytes and thus plays a negligible role in regulating hepatocyte function in uninjured livers. However, work by us and others challenges this concept. As summarized in 2 recent reviews (13, 14), profound dysregulation of fatty acid, cholesterol, bile acid, and glucose metabolism ensues when the Hedgehog pathway is disrupted in hepatocytes. Furthermore, our recent analysis of hepatocyte RNA-Seq datasets from healthy young and old mice revealed substantial age-related differences in hepatocyte Hedgehog pathway activity. Basal Hedgehog signaling was repressed in hepatocytes from old mice. Conversely, hepatocytes from young mice had greater pathway activity and quickly became senescent when Smoothened (Smo) (an obligatory Hedgehog pathway component) was disrupted experimentally (15). Although the mechanisms involved have not yet been defined, we found that disrupting Hedgehog signaling in hepatocytes evoked multiple defects that are known to trigger cell senescence, including impaired growth factor signaling, mitochondrial dysfunction, autophagy inhibition, increased oxidative stress, DNA damage, and telomere attrition. Remarkably, this was sufficient to induce MASLD in healthy young chow-fed mice. Concomitant analysis of archived human liver samples showed that the burden of Smo-depleted hepatocytes is markedly greater in patients with MASLD than in age-matched controls and parallels senescent hepatocyte accumulation and progression to cirrhosis in people with MASLD (16). Together, these findings indicate that inhibition of Hedgehog pathway activity in hepatocytes accelerates liver aging. This suggests that Hedgehog-deficient hepatocytes may be fundamentally responsible for the maladaptive regenerative responses that drive progressive hepatic dysfunction and degeneration in MASLD.

Similar to other senescent cell types, Smo-depleted hepatocytes remain viable, but their state change induces SASPs that robustly affect their secretomes. SASPs critically shape repair responses, and cumulative aging-related changes in SASPs can progressively corrupt regenerative efforts, resulting in tissue degeneration (17). Hence, in the current study, we evaluated the hypothesis that the secretome of Hedgehog-deficient hepatocytes perpetuates senescence to drive MASLD progression. Unexpectedly, our initial efforts to profile the secreted proteome of Smo-depleted hepatocytes demonstrated striking depletion of thymidine phosphorylase (TP), a protein that is normally induced by antioxidant mechanisms and functions to inhibit senescence by preserving mitochondrial fitness and optimizing repair of damaged DNA (18). Loss-of-function mutations of *Tymp*, the human gene that encodes TP, cause a rare mitochondriopathy that is typically fatal by the third to fourth decade of life (19). Our findings identify a role for Smo in the regulation of TP and thus indicate a mechanism whereby Hedgehog signaling enables hepatic metabolic resiliency. Importantly, the results demonstrate that perturbations of these basal defenses accelerate liver aging and promote the pathogenesis and progression of MASLD.

## Results

### Hepatocyte-specific deletion of Smo exacerbates MASLD in mice.

**Figure 1:**
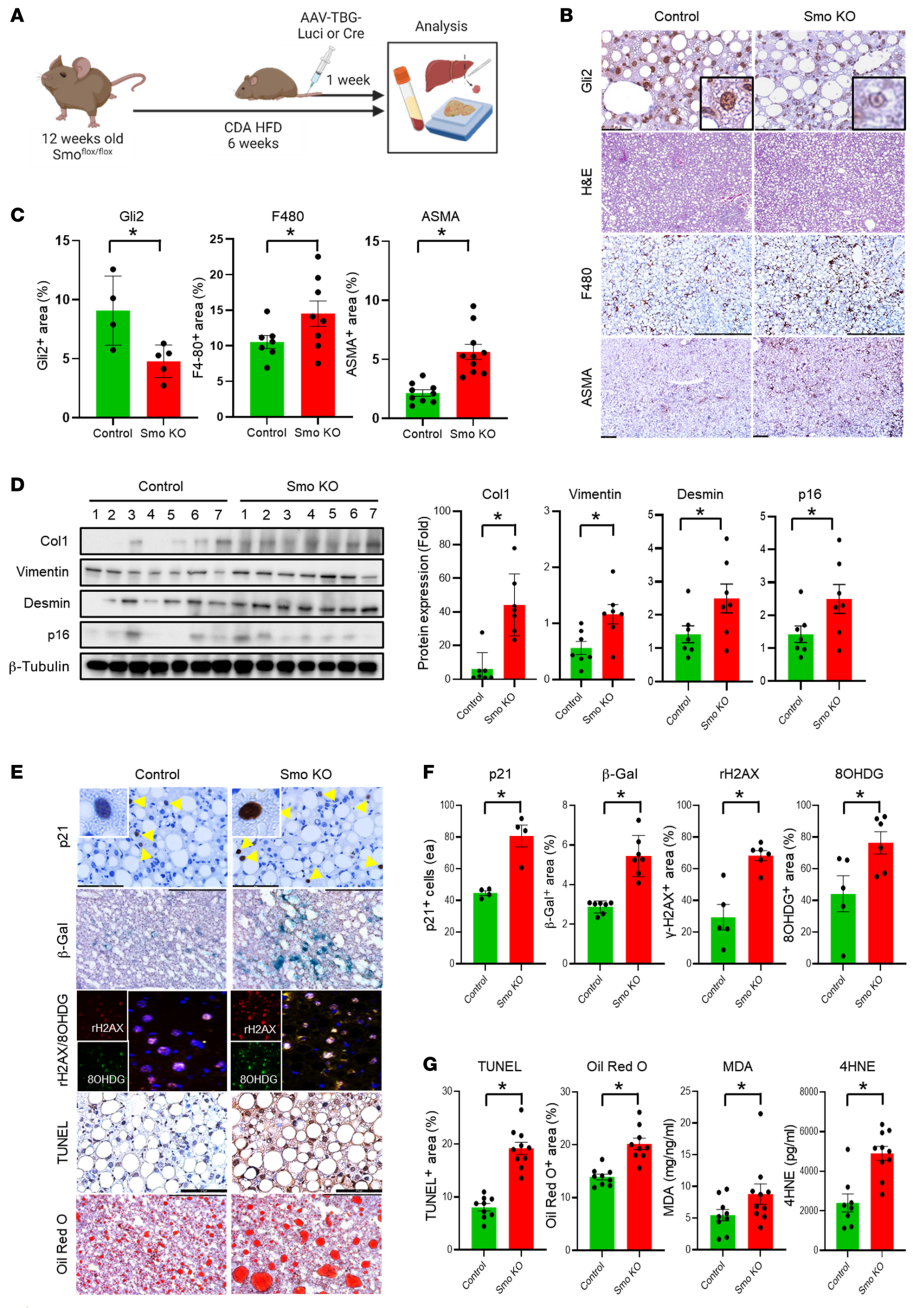
Hepatocyte-specific depletion of Smo (Smo-KO) exacerbates MASLD. (**A**) Smo^fl/fl^ mice were fed a CDA-HFD diet for 6 weeks. Mice were intravenously injected once with AAV-TBG-Luci or Cre (*n* = 9 control mice; *n* = 10 Smo-KO mice). (**B**) Representative images of staining for Gli2, H&E, F480, and α smooth muscle actin (ASMA) and (**C**) corresponding densitometric analysis of positively stained areas. Scale bars: 100 μm; original magnification, ×100 (enlarged insets). (**D**) Expression of the fibrotic and senescence markers Col1, vimentin, desmin, and p16, as detected by immunoblotting and corresponding analysis. (**E**) Representative images of staining for p21, β-gal, γH2AX (rH2AX), 8OHDG, TUNEL, and Oil Red O and corresponding densitometric analysis of positively stained areas for (**F**) p21, β-gal, rH2AX, and 8OHDG and (**G**) TUNEL, Oil Red O, MDA, and 4HNE. Scale bars: 100 μm; original magnification, ×100 (enlarged insets). Data are graphed as the mean ± SEM. *P < 0.05, by 1-way ANOVA.

Smo is an obligatory component of the Hedgehog pathway. To determine whether (and how) changes in hepatocyte Hedgehog activity influence MASLD progression, we fed Smo^fl/fl^ mice a metabolic dysfunction–associated steatohepatitis–inducing (MASH-inducing) diet for 6 weeks and used viral vectors to delete Smo selectively in hepatocytes during the final week. Results in mice with hepatocyte-specific deletion of Smo (Smo-KO) were compared with Smo^fl/fl^ mice that were treated with control vectors (Figure 1A). Our previous work in chow-fed Smo^fl/fl^ mice demonstrated that this gene-targeting approach selectively depletes Smo in hepatocytes by approximately 90% within 48–72 hours (20). Principle component analysis of bulk RNA-Seq datasets of livers from 4 choline-deficient, amino acid–restricted high-fat diet–fed (CDA-HFD-fed) control mice and 4 CDA-HFD–fed mice with Smo-depleted hepatocytes (Smo-KO) mice confirmed clustering of transcriptomes within each experimental group and revealed substantial differences in the transcriptomes of the control livers versus Smo-KO livers (Supplemental Figure 1, A and B; supplemental material available online with this article; https://doi.org/10.1172/JCI180310DS1). Further gene set enrichment analysis (GSEA) revealed that genes related to adipogenesis and fatty acid metabolism were upregulated, whereas those involved in cholesterol homeostasis were downregulated in Smo-KO livers (Supplemental Figure 1C). Also, although serum glucose levels were similar in CDA-HFD–fed control mice and mice with Smo-depleted hepatocytes, serum insulin levels and homeostatic model assessment of insulin resistance (HOMA-IR) were substantially higher in the latter group, demonstrating that deletion of Smo in hepatocytes promoted insulin resistance (Supplemental Figure 1D).

Activation of Smo is known to stabilize Gli2 protein and enable nuclear accumulation of this transcription factor (21). Consistent with this, we found that deleting Smo in hepatocytes decreased nuclear accumulation of Gli2 in CDA-HFD–fed mice (Figure 1, B and C). Mice with decreased hepatocyte Hedgehog signaling also exhibited increased hepatic inflammation (e.g., F480) and fibrosis (e.g., ASMA, Col1, vimentin, and desmin) (Figure 1, B–D). We reported previously that the severity of fibrosing steatohepatitis parallels hepatic expression of the senescence markers p16, p21, and β-gal activity in people with MASLD (15, 16), and all of these markers were increased by deleting Smo in mouse hepatocytes during CDA-HFD exposure (Figure 1, D–F). The increased senescent cell burden likely reflects exacerbated DNA damage in Smo-depleted hepatocytes because accumulation of rH2AX and 8-hydroxy-2′-deoxyguanosine (8OHDG) also increased in hepatocyte nuclei. In addition, disruption of hepatocyte Hedgehog signaling increased other markers of hepatic lipotoxicity, as evidenced by quantitative histochemistry for TUNEL and steatosis (Oil Red O), as well as increased lipid peroxidation markers (malondialdehyde [MDA] and 4-hydroxynonenal [4HNE]) in liver and serum (Figure 1, E–G). Together, these results indicate that disruption of hepatocyte Hedgehog signaling lowers the threshold for lipotoxicity, accelerates senescence, and exacerbates fibroinflammatory responses in liver cells, which, together, result in maladaptive repair and promote MASLD progression.

### The hepatocyte Smo-KO secretome is depleted of factors that promote antioxidant defense.

**Figure 2:**
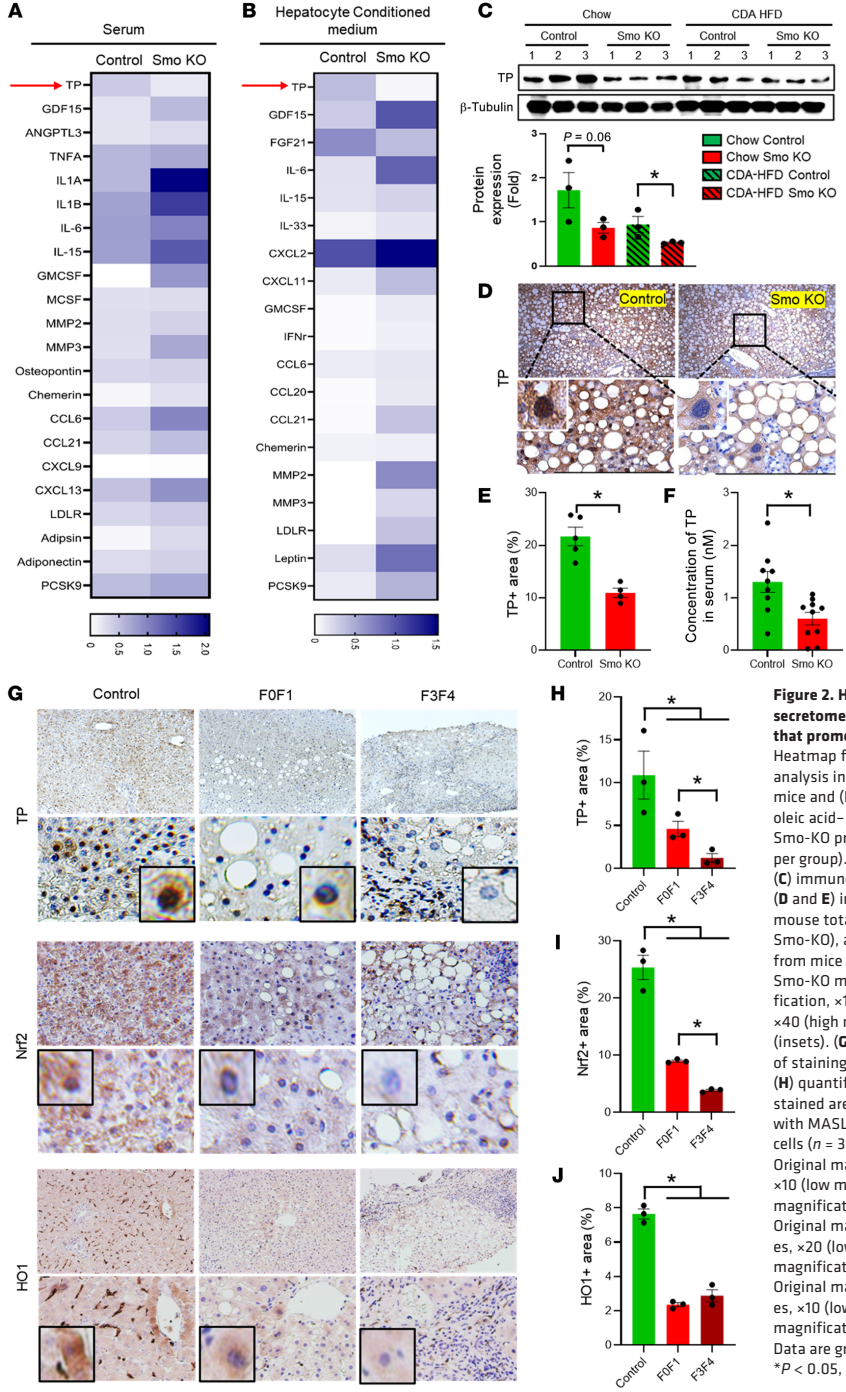
Hepatocyte Smo-KO secretome is depleted of factors that promote antioxidant defense. Heatmap from proteome profiler analysis in (**A**) serum of Smo-KO mice and (**B**) conditioned medium of oleic acid– and palmitic acid–treated Smo-KO primary hepatocytes (*n* = 3 per group). TP protein expression by (**C**) immunoblotting (*n* = 3 per group), (**D** and **E**) immunohistochemistry of mouse total liver (*n* = 5 controls, *n* = 4 Smo-KO), and (**F**) ELISA using serum from mice (*n* = 9 control mice; *n* = 10 Smo-KO mice). (**D**) Original magnification, ×10 (low magnification), ×40 (high magnification), and ×100 (insets). (**G**) Representative images of staining for TP, Nrf2, and HO1 and (**H**) quantification of the positively stained areas in cells from patients with MASLD versus healthy control cells (*n* = 3 individuals per group). (**G**) Original magnification for TP images, ×10 (low magnification), ×40 (high magnification), and ×100 (insets). Original magnification for Nrf2 images, ×20 (low magnification), ×40 (high magnification), and ×100 (insets). Original magnification for HO1 images, ×10 (low magnification), ×40 (high magnification), and ×100 (insets). Data are graphed as the mean ± SEM. *P < 0.05, by 1-way ANOVA.

During the senescence process, cells acquire various SASPs that shape regenerative responses (22). Our data indicate that deleting Smo in hepatocytes corrupted regeneration in injured livers. To identify differentially induced or repressed proteins that might mediate these maladaptive repair responses, we used a commercially available platform (Proteome Profiler Mouse Cytokine Array) to compare the secretomes in sera and hepatocyte-conditioned medium from chow-fed Smo-KO and control mice. The levels of various cytokines (e.g., TNF-α, IL-1A, IL-1B, IL-6, IL-15, IL-33, GMCSF, MCSF, and IFN-γ) were substantially higher in sera and/or conditioned medium from the Smo-KO groups compared with levels in the control groups. Interestingly, the expression of 1 protein, TP, was maximally downregulated in both the sera and conditioned medium from the Smo-KO group relative to the control groups (Figure 2, A and B). TP is a proangiogenic factor that promotes mitochondrial fitness, reduces oxidant stress, and inhibits senescence (23). Loss-of-function polymorphisms in *Tymp* (the gene that encodes TP) cause mitochondrial neurogastrointestinal encephalomyopathy (MNGIE) syndrome, a rare human mitochondriopathy that is typically fatal by mid-adulthood (24). Because our current work demonstrate that Smo depletion caused mitochondrial dysfunction, oxidative stress, DNA damage, and senescence, we elected to determine whether TP depletion has a role in the phenotype of mice with Smo-depleted hepatocytes.

Immunoblot analysis demonstrated that TP protein expression was highest in chow-fed control livers, declined in livers of CDA-HFD–fed mice with MASLD, and was lower in Smo-KO livers than was seen in the respective controls both before and during CDA-HFD exposure (Figure 2C). Immunohistochemical analysis demonstrated that TP localized in both hepatocyte cytoplasm and nuclei in control livers and was markedly depleted in both compartments in Smo-KO livers (Figure 2, D and E). Serum levels of TP were also lower in Smo-KO mice than in controls (Figure 2F).

To assess MASLD-related changes in human hepatocyte expression of *SMO* and *TYMP*, we reanalyzed publicly available single-nucleus RNA-Seq (snRNA-Seq) datasets from 2 control human livers and 2 human livers with advanced MASLD (GSE174748). Our previous deconvolution of bulk liver RNA-Seq data from a large cohort of patients with MASLD (GSE213623) indicated that hepatocyte Smo expression inversely correlates with MASLD severity in people (16). The present snRNA-Seq analysis confirmed that the proportion of Smo^+^ cells was lower in the MASLD liver hepatocyte population than in control livers (Supplemental Figure 2A). *TYMP*-expressing hepatocytes were also less abundant in the MASLD livers than in control livers in this snRNA-Seq dataset (Supplemental Figure 2A). To localize the expression of TP protein, we performed immunostaining in a representative subset of the human livers that had been processed to generate our previously published bulk liver RNA-Seq data (GSE213623) (16). As observed in the mouse livers (Figure 2, C–F), the nuclei and cytosol of a majority of hepatocytes stained strongly for TP in control human livers, and TP protein expression decreased in hepatocytes as the severity of MASLD fibrosis increased (Figure 2, G and H). Together, these findings support the concept that livers with worse MASLD have fewer TP-expressing hepatocytes. However, serum levels of TP protein were not decreased, and whole liver expression of *TYMP* mRNA was upregulated in patients with MASLD relative to controls (Supplemental Figure 2, B and D). These discrepancies may reflect cell-type–specific disease-related differences in *TYMP* expression because liver macrophage populations expressed *TYMP*, and the relative abundance of these cells increased in MASLD (data not shown).

**Figure 3:**
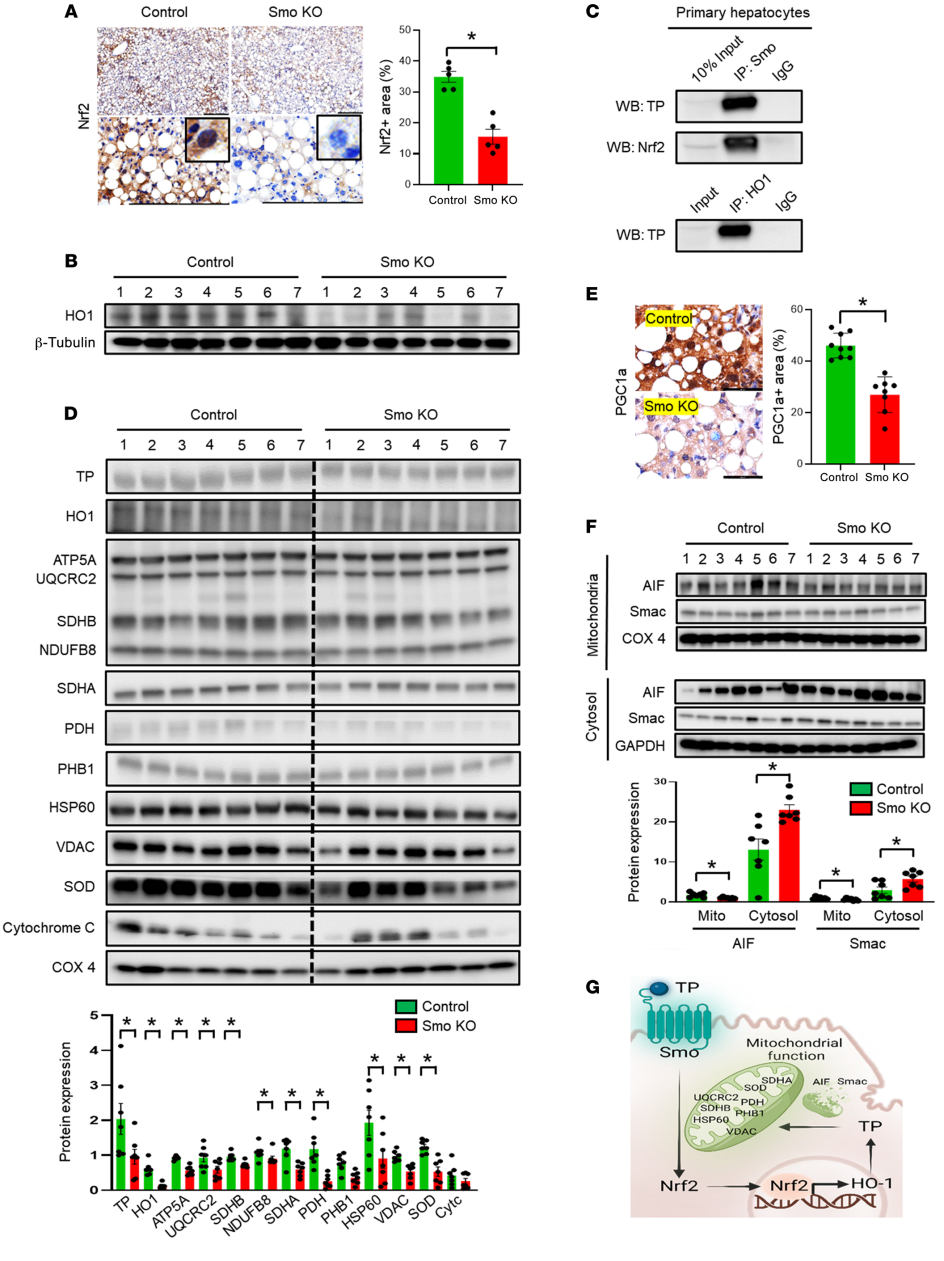
Hepatocyte Smo-KO induces mitochondrial dysfunction. (**A**) Representative images of staining for Nrf2 and quantification of the positively stained areas in liver tissues from Smo-KO versus control mice (*n* = 5 mice per group). Scale bars: 250 μm. Original magnification, ×100 (enlarged insets). (**B**) HO1 protein expression in total liver from Smo-KO versus control mice, as determined by immunoblotting (*n* = 7 mice per group). (**C**) Interaction between TP, Smo, Nrf2, and HO1 in mouse primary hepatocytes by immunoprecipitation. WB, Western blot. (**D**) Protein expression of TP, HO1, OXPHOS complexes, SDHA, PDH, PHB1, HSP 60, VDAC, SOD, and cytochrome C by immunoblotting of isolated mitochondria in total liver from Smo-KO mice (*n* = 7 mice per group). (**E**) Representative images of staining for PGC1a and quantification of the positively stained areas in liver tissues from Smo-KO versus control mice (*n* = 9 control mice; *n* = 10 Smo-KO mice). Scale bars: 60 μm. (**F**) Immunoblots showing AIF and Smac protein expression in mitochondria or cytoplasm in total liver from Smo-KO and control mice (*n* = 7 mice per group). *P < 0.05, by 1-way ANOVA. Data are graphed as the mean ± SEM. (**G**) Hypothetical design.

TP inhibits oxidant stress, and its expression is upregulated when antioxidant defense mechanisms are induced by nuclear factor erythroid 2-related factor 2 (Nrf2), a master transcriptional regulator of antioxidant defense responses (25). The responsible mechanisms remain unclear, however, as the Tymp promoter lacks obvious Nrf2-binding sites. In a previously reported study of lung cancer cells (26), the levels of TP protein were increased by overexpressing HO1, the protein encoded by Hmox1, a direct transcriptional target of Nrf2. However, in those studies, deletion of HO1 only partially diminished the positive effects of Nrf2 on TP expression, while dose-related increases in TP expression resulted when the cells were treated with *N*-acetyl cysteine. The researchers concluded that yet-to-be-determined Nrf2-sensitive antioxidant factors (including, but not limited to, HO1) promote TP accumulation. To determine whether or how the Nrf2/HO1/TP axis is affected in MASLD, we leveraged both the Duke MASLD patient bulk RNA-Seq dataset (GSE213623) and the publicly available snRNA-Seq dataset (GSE174748) to compare expression levels of *NFE2L2* (the gene that encodes Nrf2) and *HMOX1* in whole liver tissue and hepatocyte populations of human livers with and without MASLD, respectively. Expression levels of NFE2L2 and HMOX1 were decreased in both liver tissues and hepatocytes in MASLD livers (Supplemental Figure 2, A and D). We also used quantitative immunohistochemistry and Western blotting to determine how changes in TP protein relate to changes in protein levels of Nrf2 and HO1 and found that changes in hepatocyte accumulation of Nrf2 and HO1 parallel changes in TP, i.e., levels of all 3 proteins decrease with increasing severity of MASLD in humans (Figure 2, G–J) and mice (Figure 2, C–F, and Figure 3, A and B).

### Impaired mitochondrial fitness in Smo-KO hepatocytes with decreased TP, Nrf2, and HO1.

Coordinated expression of TP, Nrf2, and HO1 proteins is not a unique attribute of hepatocytes, as this was demonstrated previously in cancer cell lines (26). In many cancers, TP is overexpressed, and loss of TP promotes mitochondrial dysfunction and oxidant stress (27). Therefore, we asked whether TP, Nrf2, and HO1 might be involved in the mitochondrial integrated stress response (ISR_mt_) in hepatocytes. The ISR_mt_ optimizes mitochondrial fitness by inducing adaptive mechanisms to mitigate stress at the cellular level. During the ISR_mt_, damaged mitochondria release factors that trigger retrograde signaling to the nucleus to induce the transcription of nuclear genes that encode mitokines, such as growth/differentiation factor 15 (GDF15). The resultant increases in serum levels of GDF protein are used clinically as predictive biomarkers of morbidity and mortality in mitochondrial diseases, including those that induce mitochondrial DNA damage by disrupting the thymidine salvage pathway (28). Because GDF15 mRNA and protein levels are tightly coupled to the intensity of the ISR, we predicted that decreased accumulation of proteins that limit oxidative stress–related damage to mitochondria would amplify the upregulation of GDF15. Analysis of the snRNA-Seq dataset showed that MASLD livers tended to accumulate more GDF15^+^ hepatocytes than did healthy livers (Supplemental Figure 2A). Furthermore, the bulk liver RNA-Seq data from the large MASLD cohort, as well as the O-link proteomics analysis of sera from that cohort, indicate that the levels of GDF15 mRNA and protein increase with the severity of MASLD fibrosis in humans (Supplemental Figure 2, C and D). Importantly, the levels of GDF15 protein were also increased in conditioned medium from Smo-KO hepatocytes and sera from Smo-KO mice (Figure 2, A and B), suggesting that Smo-depleted hepatocytes with the ISR_mt_ are an important source of GDF15 in MASLD.

Although the molecular mechanisms whereby Smo might regulate the ISR_mt_ remain to be determined, interactions between the Hedgehog pathway and Nrf2 are well documented. For example, Nrf2 has been reported to activate transcription of the gene that encodes Sonic hedgehog ligand in liver cancer stem cells and replenishing Shh restores Hedgehog signaling and reestablishes the stem cell phenotype in Nrf2-depleted cells (29). Interestingly, Marin-Hurtado and colleagues demonstrated that deletion of the *Nfe2l2/Nrf2* gene in fibroblasts compromises their antioxidant defenses and suppresses the expression of multiple genes that encode cilia-associated factors, including components of the Hedgehog pathway, leading to both impaired ciliognesis and decreased Hedgehog signaling (30). Recent studies in astrocytes also demonstrate that cilia homeostasis is disrupted by mitochondrial dysfunction and resultant oxidant stress, leading the authors to propose that ciliary signaling is part of the ISR_mt_ in those cells (31). The aggregate findings support other evidence that Hedgehog pathway activity is mitoprotective (32) and suggest that Nrf2-sensitive factors may be involved. Indeed, Hedgehog signaling is activated when HO1 is overexpressed in cancer cells (33), and, as noted above, enforcing HO1 expression promotes rapid and progressive accumulation of TP protein (26). Our data show that deletion of Smo both disrupted Hedgehog signaling and reduced the accumulation of TP, HO1, and Nrf2. Therefore, to identify mechanisms whereby Smo might enhance the accumulation of these proteins, we performed immunoprecipitation experiments in extracts from primary hepatocytes. Remarkably, we found that Smo, TP, Nrf2, and HO1 physically interacted (Figure 3C). This finding suggests that Smo might be involved in posttranscriptional mechanisms that enable cellular accumulation of factors that critically mediate antioxidant defense and, conversely, that factors that promote antioxidant defense help to maintain Hedgehog pathway integrity and thus, ciliary homeostasis.

TP maintains nucleoside pools for mitochondrial DNA synthesis, and, thus, depleting TP compromises the repair of mitochondrial DNA and leads to mitochondrial dysfunction and increased mitochondrial ROS production (34). To determine whether Smo-depleted hepatocytes that are unable to form complexes of TP-Nrf2-HO1 exhibit impaired mitochondrial fitness, we isolated mitochondria from livers of CDA-HFD–fed Smo-KO and control mice and performed immunoblot analysis for TP, Smo, Nrf2, HO1, and OXPHOS components as well as other mitochondrial markers. Deletion of Smo decreased mitochondrial accumulation of TP and HO1 and markedly downregulated OXPHOS complexes 2, 3, and 5, as well as mitochondrial markers (e.g., succinate dehydrogenase complex flavoprotein subunit A, [SDHA], pyruvate dehydrogenase complex [PDH], prohibitin 1 [PHB1], heat shock protein 60 [HSP60], voltage-dependent anion channel [VDAC], and superoxide dismutase [SOD]) (Figure 3D). Smo and Nrf2 proteins were not detected in mitochondria of Smo-KO or control mice. However, expression of the nuclear DNA-encoded mitochondrial biogenesis marker and Nrf2-target gene, peroxisome proliferator-activated receptor γ coactivator 1a (PGC1a), was substantially decreased in the CDA-HFD Smo-KO livers (Figure 3E), and nuclear accumulation of Nrf2 was markedly decreased in hepatocytes of these mice (Figure 3A). In addition, the Smo-KO group had lower mitochondrial levels of apoptosis-inducing factor (AIF) and second mitochondria-derived activator of caspase (Smac) but higher cytosolic levels of these proteins (Figure 3F), consistent with increased mitochondrial membrane permeability and activation of mechanisms that promote apoptosis. Hence, the collective data suggest a conceptual model for maintenance of hepatocyte resiliency, whereby Smo, an obligatory signaling component of the Hedgehog pathway, regulates the bioavailability of ISR_mt_-related antioxidant proteins to maintain mitochondrial fitness and assure hepatocyte metabolic flexibility and viability (Figure 3G). Therefore, depletion of Smo is predicted to enhance hepatocyte susceptibility to lipotoxicity but limit hepatocyte regenerative capacity and thus exacerbate MASLD.

### TP protects hepatocyte cultures from lipotoxicity and senescence.

Lipotoxicity promotes senescence, and accumulation of senescent hepatocytes parallels the progression of liver damage in MASLD. To determine whether TP directly influences hepatocyte susceptibility to senescence, we treated AML-12 cells (a mouse hepatocyte cell line) with palbociclib (a selective inhibitor of the cyclin-dependent kinases CDK4 and CDK6) for 5 days to induce senescence and then supplemented the culture medium with either recombinant TP protein or vehicle for an additional 48 hours (Supplemental Figure 3A). Results in AML-12 cells that had been cultured in palbociclib-supplemented medium for 7 days were compared with cultures of AML-12 cells that had been maintained in normal growth-promoting medium for the same duration. As expected, cultures treated with palbociclib and vehicle had substantially fewer hepatocytes than control cultures. Remarkably, treating palbociclib cultures with TP restored cell numbers to levels of control cultures that had not been exposed to palbociclib (Supplemental Figure 3B). Also, compared with control cultures, palbociclib cultures that were treated with vehicle exhibited increased steatosis (Oil Red O), lipotoxicity (4HNE), and senescence (β-gal). Treating palbociclib cultures with TP substantially reduced Oil Red O staining, 4HNE, and β-gal activity (Supplemental Figure 3, B and C). Together, these data show that TP acted directly on hepatocytes to inhibit lipotoxicity and senescence.

### Manipulation of TP in cultured hepatocytes modulates antioxidants and susceptibility to lipotoxicity and senescence.

Next, we tested the effects of TP in an in vitro model of hepatocyte lipotoxicity, in which Huh7 cells were cultured with oleate and palmitic acid (OPA) for a total of 4 days. During the last 2 days of culturing under these lipotoxic conditions, half the cultures were treated with recombinant TP protein and half were treated with a pharmacologic TP inhibitor (TPI). Results in the OPA (lipotoxic) cultures were compared with Huh7 cells that had been cultured for 4 days in normal growth medium (Supplemental Figure 4A). As expected, the OPA cultures had substantially more steatosis (Oil Red O) and senescence (β-gal activity) than did control cultures (Supplemental Figure 4B). Relative to the control cultures, the OPA cultures also showed decreased expression of Nrf2 and HO1 and increased expression of p21 (Supplemental Figure 4C), supporting the concept that lipotoxic cultures had decreased antioxidant defense and increased cell-cycle arrest. Remarkably, treating lipotoxic cultures with recombinant TP protein reduced Oil Red O staining and β-gal activity, increased Nrf2 and HO1, and decreased p21, while inhibiting TP had the opposite effect on each of these parameters (Supplemental Figure 4, B and C). Hence, the collective results of these studies in Huh7 cells complement and extend the studies in AML-12 cells and, together, provide strong evidence that TP can act directly on hepatocytes to maintain their resiliency during lipotoxic stress by boosting antioxidant defenses that inhibit lipotoxicity and senescence.

### Replenishing TP restores mitochondrial fitness and rescues Smo-depleted hepatocytes from lipotoxicity and senescence.

**Figure 4:**
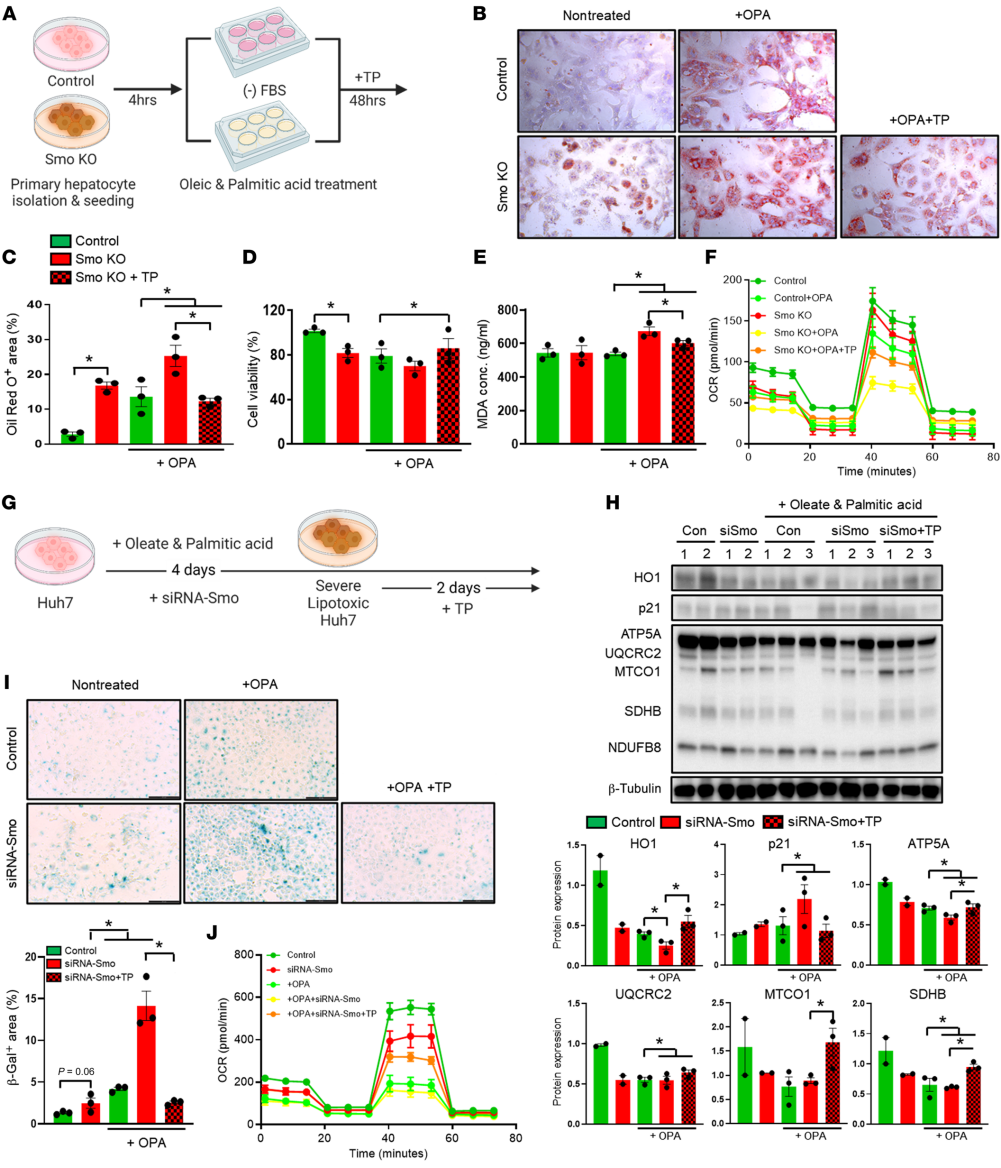
Replenishing TP restores mitochondrial fitness and rescues Smo-depleted hepatocytes from lipotoxicity and senescence. (**A**) Experimental scheme. (**B** and **C**) Oil Red O staining and (**D**) percentage of Smo-KO primary hepatocytes by CCK-8 assay. Original magnification, ×200. (**E**) MDA concentration (conc.) in conditioned media of Smo-KO primary hepatocytes. (**F**) OCR using the Seahorse extracellular flux analyzer in Smo-KO primary hepatocytes. (**G**) Experimental scheme. (**H**) Protein expression by immunoblot assay and corresponding morphometric quantification in siRNA-Smo–transfected Huh7 cells. (**I**) β-Gal staining and corresponding morphometric quantification in siRNA-Smo–transfected Huh7 cells. Scale bars: 131.4 μm. (**J**) OCR of siRNA-Smo–transfected Huh7 cells using the Seahorse extracellular flux analyzer. Data from triplicate experiments are graphed and are shown as the mean ± SEM. *P < 0.05, by 1-way ANOVA.

TP production was reduced in Smo-KO hepatocytes (Figure 2, B and C), and mice with Smo-depleted hepatocytes developed worse MASH when challenged with diets that promoted hepatic lipotoxicity (Figure 1). To determine whether treating Smo-KO hepatocytes with TP could protect them from lipotoxicity, we isolated primary hepatocytes from control and Smo-KO mice and cultured the cells in OPA-enriched, serum-depleted medium that was Supplemented with vehicle or recombinant TP protein for 48 hours. Results in the OPA cultures were compared with cells cultured in serum-depleted medium without OPA (Figure 4A). As expected, culturing in OPA-enriched medium increased steatosis (Oil Red O) in both control and Smo-KO hepatocytes, and Smo-KO cells were more steatotic than control cells under both culture conditions (Figure 4, B and C). Although the numbers of hepatocytes were similar in Smo-KO and control OPA cultures (Figure 4D), only Smo-KO cultures demonstrated enhanced accumulation of oxidized lipids (malondialdehyd [MDA]) when exposed to OPA (Figure 4E). Treatment of Smo-KO cultures with TP completely protected them from the steatosis-inducing effects of OPA (Figure 4, B and C), prevented them from OPA-induced accumulation of MDA (Figure 4E), and increased cell numbers by approximately 10% despite the serum-depleted culture conditions (Figure 4D). Evidence that replenishing TP can rescue Smo-depleted hepatocytes from lipotoxicity indicates that TP operates downstream of Smo to protect hepatocytes from lipid-related stress and supports the concept that Smo mainly enhances TP stability, although more research is needed to validate this hypothesis. To determine how these Smo/TP-sensitive differences in lipotoxicity relate to differences in mitochondrial fitness, experiments were repeated and Seahorse analysis was done to compare mitochondrial oxygen consumption rates (OCRs) (Figure 4F). The OCR in Smo-KO hepatocytes was lower than in controls at the basal level. Although OPA suppressed these functions in both control and Smo-KO hepatocytes, OPA-treated Smo-KO hepatocytes also demonstrated the lowest OCRs when cultured in lipotoxic conditions. Importantly, replenishing TP partially protected Smo-KO hepatocytes from OPA-induced mitochondrial dysfunction and substantially improved the OCR in Smo-KO hepatocytes during exposure to lipotoxic stress (Figure 4F). To verify and further clarify the effects of TP during Smo gene disruption, we transfected Huh7 cells with siRNA-Smo and cultured these cells in OPA-enriched medium for 4 days, adding either vehicle or recombinant TP protein for the final 2 days of culturing (Figure 4G). As noted in our earlier studies using Smo-KO mice (Figure 1) (16), disrupting the Smo gene in hepatocytes is sufficient to induce senescence basally and dramatically exacerbates senescence (β-gal activity) that is provoked by lipotoxic stress (induced here by culturing in OPA-enriched medium) (Figure 4I). Remarkably, TP treatment completely protected siRNA-Smo–treated Huh7 cells from OPA-induced senescence, as evidenced by reduced β-gal staining (Figure 4I) and decreased expression of the cell-cycle inhibitor p21 on immunoblots (Figure 4H). These changes in hepatocyte senescence were reciprocally related to changes in accumulation of HO1 and mitochondrial OXPHOS complexes (Figure 4H), suggesting that TP treatment reversed depletion of the Smo-regulated factors that maintain mitochondrial fitness in metabolically stressed hepatocytes. Consistent with this concept, Seahorse analysis showed that Smo siRNA decreased the OCR in Huh7 cells and demonstrated that this parameter was more sensitive to OPA suppression in Smo-depleted cultures than in Huh7 controls. Importantly, similar to Smo-KO primary hepatocytes (Figure 4F), Smo-depleted Huh7 cells were rescued from the mitoinhibitory effects of OPA by TP treatment (Figure 4J).

### Inhibition of TP exacerbates MASH and reduces antioxidant defense and mitochondrial fitness in livers of CDA-HFD mice.

**Figure 5:**
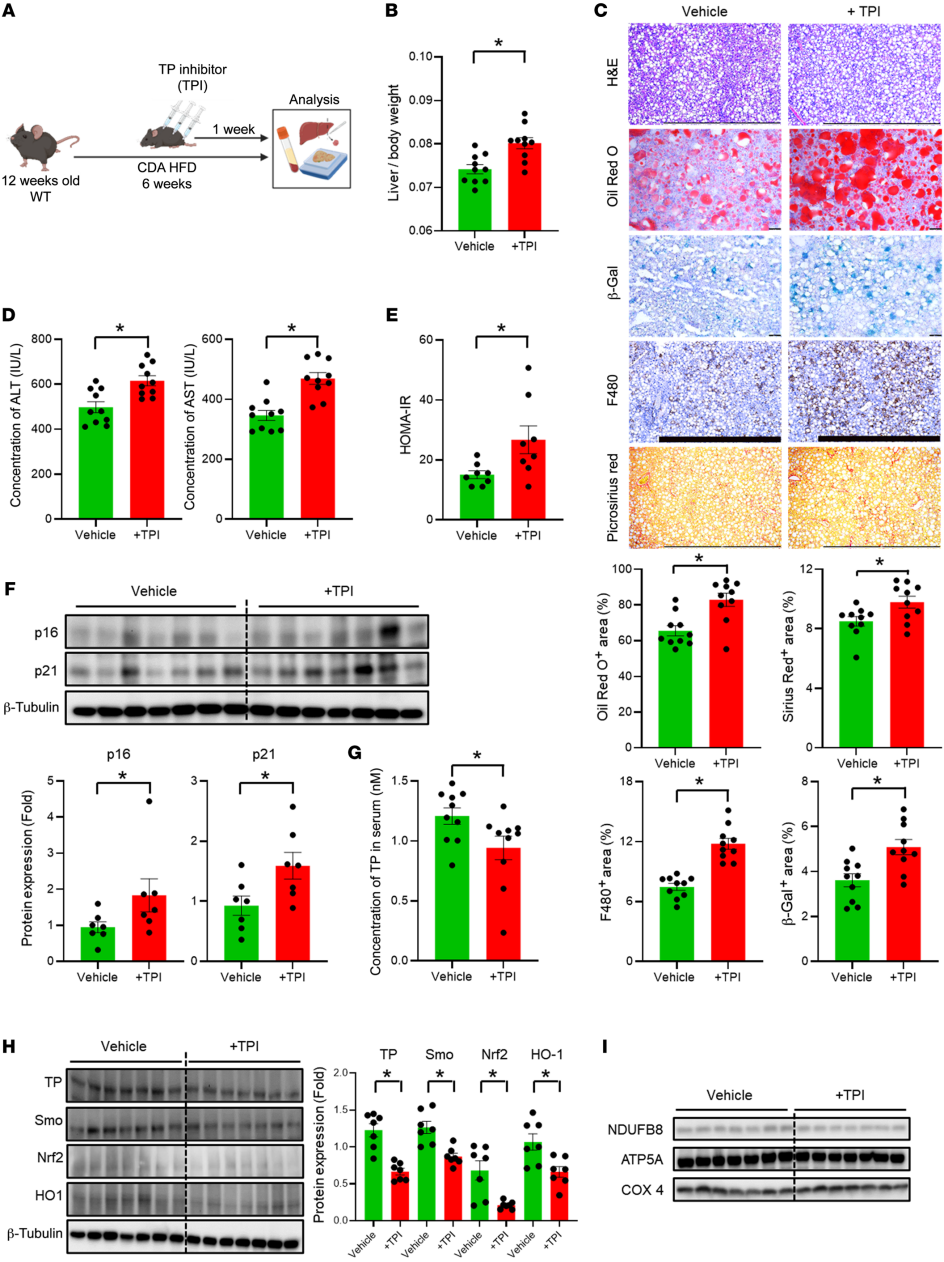
Inhibiting TP exacerbates diet-induced MASH and liver fibrosis in WT mice. (**A**) WT mice were fed a CDA-HFD diet for 6 weeks and were intraperitoneally injected with a TPI (tipiracil-HCl) or its vehicle 3 times. (**B**) Liver/body weight ratio in TPI-treated versus vehicle-treated mice (*n* = 10 mice per group). (**C**) Representative images of staining for H&E, Oil Red O, Picrosirius red, F480, β-gal and corresponding densitometric analysis of positively stained areas. Scale bars: 100 μm (H&E-, F480-, and Picrosirius red–stained images) and 50 μm (Oil Red O– and β-gal–stained images). Serological results of hepatic function markers (**D**) ALT and AST and (**E**) HOMA-IR (*n* = 10 mice per group). (**F**) Expression of the senescence markers p16 and p21 detected in total liver from TPI-treated mice, as detected by immunoblotting (*n* = 7 mice per group). (**G**) Concentration of TP in serum from vehicle- and TPI-treated mice (*n* = 10 mice per group). (**H**) Protein expressions of TP, Smo, Nrf2, and HO1 detected in total liver from TPI-treated mice, as detected by immunoblotting (*n* = 7 mice per groups). (**I**) Expression of the mitochondrial OXPHOS complex markers NDUFB8 and ATP5A detected in total liver mitochondria from vehicle- or TPI-treated mice, as detected by immunoblotting (*n* = 7 mice per group). Data are graphed as the mean ± SEM. *P < 0.05, by 1-way ANOVA.

We used adenoviral vectors to disrupt Smo in hepatocytes of Smo^fl/fl^ mice, as both viral vectors and gene manipulation can have off-target effects. Therefore, to assure that the responses we observed in Smo-KO mice resulted from a decreased abundance of TP, we fed WT a CDA-HFD for 6 weeks to induce MASH and treated them with vehicle or TPI during the final week of the diet (Figure 5A). TPI had no effect on body weight but markedly increased liver weight and thus the liver/body weight ratio (Supplemental Figure 5, A and B, and Figure 5B). WT mice treated with TPI during the final week of CDA-HFD administration also showed hyperglycemia, hyperinsulinemia, and increased HOMA-IR at sacrifice (Supplemental Figure 5C and Figure 5E). Thus, similar to Smo-KO mice, TPI-treated WT mice developed systemic insulin resistance when fed a CDA-HFD. Insulin resistance is thought to play an important role in MASLD pathogenesis. Consistent with this, hepatic steatosis was increased in TPI-treated mice as visualized on both H&E- and Oil Red O–stained liver sections (Figure 5C), confirming other evidence which showed that inhibiting TP promotes steatosis in cultured hepatocytes (Supplemental Figures 3 and 4). TPI-treated mice also had higher serum levels of aspartate aminotransferase (AST) and alanine aminotransferase (ALT) (Figure 5D), consistent with the results of our hepatocyte culture studies, which showed that TP directly regulated hepatocyte susceptibility to lipotoxicity (Supplemental Figure 3). Lipotoxicity induces senescence in cultured hepatocytes, and TP regulates this process (Supplemental Figure 4). Furthermore, senescent hepatocytes incite liver inflammation and fibrosis (35). Consistent with these facts, livers of TPI-treated mice accumulated more senescent cells (evidenced by greater β-gal activity and increased expression of p16 and p21) (Figure 5, C and F). They also exhibited greater inflammation (F480 staining, Figure 5C) and worse fibrosis, as evidenced by increased expression of fibrosis markers (vimentin and desmin) on immunoblots (Supplemental Figure 5D) and more collagen fibrils on Sirius red-stained liver sections (Figure 5C). Together, these results demonstrate that inhibition of TP exacerbates diet-induced MASH in mice by promoting liver lipotoxicity and senescence.

Senescence can be triggered by chronic mitochondrial damage and resultant oxidant stress. TP is important for maintaining mitochondrial fitness and forms a complex with Nrf2 and HO1 (Figure 2C), factors that have critical roles in antioxidant defense. Most important, our data reveal that Smo can physically interact with TP and its binding partners (Figure 3C). Therefore, we analyzed liver samples from vehicle and TPI-treated, CDA-HFD–fed mice to determine whether and how TPI treatment affects these relationships. We found that TPI treatment reduced both serum and liver levels of TP (Figure 5, G and H). Liver levels of Nrf2, HO1, and Smo proteins were also substantially reduced in the TPI-treated group (Figure 5H), demonstrating that inhibition of TP recapitulates the liver phenotype of mice with Smo-depleted hepatocytes and supporting the concept that TP is needed to stabilize these proteins and maintain proper antioxidant defense. Indeed, TPI treatment depleted components of OXPHOS complexes (NDUFB8 and ATP5A) in mice fed MASH-inducing diets (Figure 5I and Supplemental Figure 5, E and F). Importantly, GDF15 protein levels were also increased in hepatocytes of TPI-treated mice (Supplemental Figure 5G), suggesting that inhibition of TP induced ISR_mt_ in hepatocytes, leading to the exacerbation of MASLD.

## Discussion

We recently discovered that adult livers rely on the Hedgehog pathway to shield hepatocytes from lipotoxicity and suggested that this slows their biological aging by enabling metabolic flexibility that is necessary to maintain liver homeostasis and prevent MASLD (15, 16, 20). These concepts have not been widely accepted despite growing evidence that hepatocyte Hedgehog signaling broadly regulates metabolism and regenerative capacity in these cells (13, 14). Thus, the current studies are important because they provide additional proof that the Hedgehog pathway critically controls the liver aging process and, hence, susceptibility to MASLD. Furthermore, we have now identified a Smo-dependent factor, TP, that has a major role in preventing hepatocyte aging and showed that this benefit accrues because TP promotes mitochondrial fitness. Although more research is needed to delineate the mechanisms involved, TP is known to facilitate the repair of mitochondrial DNA, and this reduces the risk for mitochondrial dysfunction and oxidative stress (24). We have shown that Smo also promoted coincident accumulation of Nrf2 and HO1, two other proteins involved in antioxidant defense (30) and demonstrated that all 4 proteins directly interacted. More research is needed to determine whether(and how) these complexes control protein stability and/or localization, but our data show that deletion of Smo blocked nuclear localization of Nrf2 and reduced mitochondrial accumulation of TP and HO1, whereas inhibition of TP decreased Smo accumulation and recapitulated all the negative consequences of hepatocyte Smo depletion, including MASLD and insulin resistance.

The findings suggest a self-reinforcing mechanism, whereby Hedgehog signaling promotes the accumulation of TP and, in turn, TP promotes the accumulation of Smo to sustain Hedgehog signaling. Hedgehog signaling is coupled to primary cilia, a key nutrient-sensing organelle, by bidirectional signaling: Hedgehog pathway components traffic on and off primary cilia to control Hedgehog signaling; conversely, ciliogenesis itself is regulated by Hedgehog pathway activity (36). As mentioned earlier, the significance of cilia (and by extension, Hedgehog signaling) in adult hepatocytes has been debated because cilia have been observed in fewer than 10% of hepatocytes at a given time point (12, 37). However, more research is needed to clarify if and how cilia might mediate the striking phenotype that results when Smo is depleted in these cells. The issue is quite complicated for 3 main reasons. First, cilia are dynamic structures, and they are much smaller in hepatocytes than in cholangiocytes (12). Therefore, it may simply be easier to identify cholangiocyte cilia than hepatocyte cilia, leading to an underestimation of ciliary abundance in hepatocytes. Second, hepatocytes and cholangiocytes themselves exhibit significant plasticity — each cell type can transition to become the other cell type and then revert back to its original phenotype (38). This confounds efforts to determine whether the seemingly small subpopulation of hepatocytes that express primary cilia at any time point are in the process of transitioning into cholangiocytes (or derive from cholangiocytes that have nearly become hepatocytes). Third, in either situation, cilia-linked Smo activity may persist (or emerge) even after (or before) the state transition is obvious. In any case, our data complement and extend growing evidence that the Hedgehog pathway is coupled to mitochondria, the main energy-producing organelle in cells (16). Our findings suggest that the Hedgehog pathway (i.e., Smo) links cilia to mitochondria and thus couples nutrient sensing to energy production. This is a critical insight because defective nutrient sensing is a key driver of tissue degeneration related to aging and metabolic dysfunction (39), the major risk factors for MASLD and other tissue damage associated with obesity and type 2 diabetes (40). The model also helps explain the pathobiology of inherited ciliopathies and mitochondrial diseases, both of which are characterized by dysregulated morphogenesis, progressive metabolic dysfunction, premature tissue degeneration, and decreased longevity.

The Hedgehog pathway has long been known to promote mitochondrial fitness and increase longevity in model organisms (7). Our data show that TP was readily detected in the livers of healthy people and mice. Conversely, inherited deficiency of TP causes systemic mitochondrial dysfunction, i.e., MNGIE syndrome, and substantially shortens the lifespan of individuals with this syndrome (24). TP is a key enzyme in the thymidine salvage pathway, and thus TP deficiency compromises the repair of mitochondrial DNA deletions that occur regularly in healthy cells as a consequence of routine metabolic activity. This leads to mitochondrial dysfunction that triggers the ISR_mt_. The ISR_mt_ is a dynamic and potentially progressive process that aims to “right-size” the mitochondrial network either to meet the energy demands required to optimize cell viability or to assure the elimination of terminally wounded cells to “make room” for healthier replacements so that tissue integrity is restored and organismal lifespan is preserved (41). The intensity of the ISR_mt_ is reflected by serum levels of mitokines and other factors released from cells with dysfunctional mitochondria — rising mitokine levels are indicative of an unsuccessful ISR_mt_ and, thus, persistent mitochondrial dysfunction. Hence, serum levels of the mitokine GDF15 are used clinically as a predictor of morbidity and mortality in people with mitochondrial diseases (42). Early work showed that steatotic hepatocytes in MASLD livers have mitochondrial abnormalities (43). We discovered that GDF15 production was increased in Smo-deficient hepatocytes and upregulated in the livers of people with MASLD. These results indicate that loss of Smo, and resultant decreases in TP, promoted the ISR_mt_. Thus, the findings complement and extend earlier reports showing that both Smo-depleted senescing hepatocytes and GDF15 levels increase with age in people and are particularly high in patients with advanced fibrosis related to MASLD (44).

Our comparison of conditioned medium from control and Smo-depleted hepatocytes demonstrates differences in GDF15, TP, and multiple other proteins. The relative abundance of many, but not all, of these factors differed similarly in sera collected from the respective groups of mice, suggesting that hepatocytes were major sources of most of the proteins. However, some differentially abundant proteins in sera were not identified by the hepatocyte-conditioned medium analyses. While technical issues may have contributed to these discrepancies, it is also conceivable that the differences reflected changes in the secretomes of other types of cells in liver and extrahepatic tissues that were caused by deletion of Smo in hepatocytes. Defining the mechanisms that orchestrate intercellular and interorgan crosstalk to enable systemic adaptations to metabolic stress has become a focus of research that aims to identify diagnostic, prognostic, and therapeutic targets for MASLD and other metabolic dysfunction–associated diseases. More research is needed to unravel how Smo-dependent changes in hepatocyte mitochondrial stress fit into this complex pathobiology. Single-cell and multiomics analytical approaches will likely be needed to map and integrate time-sensitive changes in multiple targets because studies of GDF15 and TP exemplify the fact that stress-related factors have pleiotropic actions that are context dependent (45).

Case series of patients with MNGIE syndrome provide the most direct evidence that loss of TP per se promotes progressive steatotic liver disease. A recent consensus conference of MNGIE experts reported hepatopathy (i.e., liver steatosis evolving to cirrhosis) in 22% of patients with MNGIE based on their meta-analysis of published literature, but this figure may underestimate the prevalence of liver dysfunction, as the authors also noted that “death is mainly due to GI and liver complications,” including “liver failure” (24). Furthermore, because the liver is a major source of circulating TP, liver transplantation has been done to treat patients with MNGIE, and these experts recommended liver replacement as a preferred treatment in selected MNGIE patients. Insulin resistance occurs in MNGIE syndrome, and disrupting Tymp blocks insulin signaling in cultured adipocytes (46), suggesting that adipocyte-related systemic metabolic dysfunction contributes to MNGI-related hepatic steatosis. However, given that gastrointestinal dysmotility (a dominant clinical feature of MNGIE syndrome) promotes intestinal dysbiosis, gut/liver axis pathobiology may also have a role in liver damage induced by TP deficiency. Interestingly, we have reported that deletion of Smo in hepatocytes dysregulates bile acids and rapidly provokes intestinal dysbiosis in mice (20). Our current studies show that Smo promoted the accumulation of TP and vice versa, raising the possibility that primary loss of either protein may trigger hepatocyte mitochondrial dysfunction which, in turn, would dysregulate bile acid homeostasis and the gut/liver axis to drive progression of steatotic liver disease. Therefore, although we found no reports linking MNGIE syndrome with Hedgehog pathway inhibition, our data clearly demonstrate that deleting Smo in hepatocytes reduced hepatic production of TP and show that loss of TP activity recapitulated the negative effects of Smo deletion on hepatocyte mitochondria, antioxidant defense, oxidative damage, lipotoxicity, and senescence. Since all these defects are reported to occur in other cell types when TP is inhibited (18) and Smo-deficient hepatocytes accumulate as MASLD progresses (16), the available data suggest that MASLD may be one facet of a MNGIE-like syndrome. This concept is supported by a recent study that used radiolabeled TP and PET to localize TP in control mice and mice with diet-induced MASLD. TP mainly accumulated in the livers of control mice, and hepatic TP content was substantially reduced in mice with MASLD (47). Thus, while seemingly heretical, the possibility that loss of Tymp/TP function promotes MASLD pathogenesis is a worthy topic for future research, given the acknowledged associations of MASLD with hepatic mitochondrial dysfunction, disordered gastrointestinal motility, sarcopenia, and neurodegenerative disorders (48).

## Methods

### Sex as a biological variable

Sex was not considered as a biological variable.

### Animal studies

#### Phenotypic comparison of secretomes from control or Smo-KO mice.

Adult male Smo tm2Amc /J (Smo^fl/fl^) mice on a C57Bl6/J background (JAX stock no. 004526; The Jackson Laboratory) were used. At 12 weeks of age, mice were injected by tail vein with 5 × 10^11^ genome equivalents of AAV8-TBG-luciferase (control) or AAV8-TBG-Cre recombinase (Smo-KO) to selectively delete Smo in hepatocytes. Vectors were obtained from the University of Pennsylvania Viral Vector Core and Addgene. Control (*n =* 4) and Smo-KO (*n =* 4) mice were fed a standard chow diet and then sacrificed 1 week after vector injection to harvest the liver and serum. Another cohort of control (*n =* 3) and Smo-KO (*n =* 3) mice underwent in situ liver perfusion to isolate hepatocytes for culture and collection of conditioned medium. Serum and hepatocyte-conditioned medium were characterized using commercial kits (see Supplemental Methods).

#### Liver perfusion and primary hepatocyte isolation.

A total of 6 Smo^fl/fl^ mice were used for these studies. After treatment with vectors (see above), primary hepatocytes were isolated from 3 control mice and 3 Smo-KO mice and cultured for up to 4 days to assess the effects of TP on susceptibility to lipotoxicity.

#### Effect of Smo-KO on MASLD.

To examine the effect of Smo on MASLD susceptibility, control (*n =* 9) and Smo-KO (*n =* 10) mice were fed a CDA-HFD diet (A06071302, choline-deficient, l-amino acid–defined diet with 60% kcal from fat; Research Diets) for 6 weeks. Vectors were injected by tail vein 1 week before sacrifice. At the end of diet administration of the CDA-HFD studies, mice were sacrificed, blood was obtained, and liver tissues were fixed in phosphate-buffered formalin for histological analysis or flash-frozen in liquid nitrogen and stored at –80°C.

#### Effect of TP on MASLD.

To study the role of TP in MASLD progression, C57BL/6J male mice (The Jackson Laboratory) were fed a CDA-HFD diet for 6 weeks and intraperitoneally injected with 1.7 mg/kg tipiracil-hydrochloride, a TP inhibitor (TPI-HCl; *n =* 10) (MedChemExpress) or its vehicle (PBS; *n =* 10) three times per week during the final week before sacrifice to obtain blood and liver specimens.

Assay details are provided in the Supplemental Methods.

### Cell culture studies

Smo and TP were manipulated in primary mouse hepatocytes and cell lines (AML-12, Huh7) to determine the effects on senescence and lipotoxicity (see the Supplemental Methods).

### Human studies

Details are provided in the Supplemental Methods.

### Statistics

Data are expressed as the mean ± SEM. Statistical significance between 2 groups was evaluated using the 1-tailed Student’s *t* test, while comparisons of multiple groups were assessed by 1-way ANOVA, followed by Student-Newman-Keul’s test. A *P* value of 0.05 or less was considered statistically significant.

### Study approval

We affirm that our research with human samples was conducted in accordance with the Declarations of Helsinki and Istanbul and approved by the Duke University Health System (DUHS) IRB (Pro00005368), with written consent given by all participants. Deidentified liver sections from patients with different stages of liver fibrosis were analyzed. Also, all animal studies were approved by the Duke University IACUC (A200-21-09) and fulfilled NIH and Duke University IACUC requirements for humane animal care.

### Data availability

All data generated by the present study are included in this article and the Supplemental data. The bulk RNA-Seq data from the CDA-HFD–fed control versus Smo-KO mice have been deposited in the NCBI’s Gene Expression Omnibus (GEO) database (GEO GSE273523). Additional public datasets for the samples discussed in the present study are available in the GEO database under the following accession numbers: GSE174748 (snRNA-Seq from 2 control and 2 advanced MASLD human) and GSE213623 (Duke MASLD cohort). Values for all data points in graphs are reported in the Supporting Data Values file.

## Author contributions

JHJ and AMD conceived of the experiments. JHJ, RKD, RMD, and SHO performed experiments. JHJ, RKD, LW, GG, JH, SSP, and AMD analyzed data. JHJ and AMD wrote the manuscript. AMD secured funding for the study. All authors reviewed and approved the manuscript.

## Supplementary Material

Supplemental data

Unedited blot and gel images

Supporting data values

## Version 1 08/27/2024

In-Press Preview

## Version 2 10/01/2024

Electronic publication
