# Supplementary material for: The senescence-associated secretome of Hedgehog-deficient hepatocytes drives MASLD progression
Source: J Clin Invest. 2024 Aug 27;134(19):e180310. doi: 10.1172/JCI180310 (PMC11444248; doi:10.1172/JCI180310)
Supplement: Supplemental data [file jci-134-180310-s006.pdf]

The Senescence-associated Secretome of Hedgehog-deficient Hepatocytes Drives MASLD Progression

Supplemental data

Ji Hye Jun[1], Kuo Du[1], Rajesh Kuma Dutta[1], Raquel Maeso-Diaz[1], Seh Hoon Oh[1], Liuyang Wang[2], Guannan Gao[3], Ana Ferreira[3], Jon Hill[3], Steven S Pullen[3], Anna Mae Diehl[1#]

# Corresponding Author:

Anna Mae Diehl, M.D. annamae.diehl@duke.edu

Synderman Building – Suite 1073

Division of Gastroenterology, Duke University

Durham, NC 27710

Disclosures: Funding from a sponsored research agreement with Boehringer-Ingelheim Pharmaceuticals helped to support this work. The authors declare no other conflicts of interest.

Author Contributions: J.H.J and A.M.D. conceived of the experiments. J.H.J, K.D., R.M.D., and S.H.O performed experiments. J.H.J, R.K.D., L.W., G.G., J.H., S.S.P., and A.M.D. analyzed data. J.H.J and A.M.D. wrote the manuscript. A.M.D. secured funding for the study. Everyone reviewed and approved the manuscript.

Grant support: RO1 DK 07794 (AMD), R56 DK134334 (AMD), AA10154 (AMD), Sponsored Research Agreement 33751(AMD) and the Duke Endowment (AMD).

Keywords: MASLD; Senescence; Hedgehog signaling; Secretome

Supplemental Methods

Human studies

Duke MASLD cohort for bulk-RNA seq analysis. We analyzed a publicly available GSE213623 RNA sequencing data set from a cohort of n = 368 liver biopsies that were classified into healthy and NASH/MASH with F0/F1, F2, or F3/F4 fibrosis. This cohort was generated from healthy obese controls (no histologic features of chronic liver disease, n = 69) or biopsy-proven MASLD (n = 299) that archived in the Duke University Health System (DUHS) MASLD Clinical Database and Biorepository. A summary of patient characteristics is detailed in **Supple Table 1**.

Single nuclei (sn)-RNA seq data analysis. Publicly available (GSE174748) single nuclei RNA seq data containing feature barcode matrices was downloaded from GEO website. Seurat (v 5.0.3) was used for filtration and for further processing of feature barcode matrices. After filtration, data were log normalized, scaled, and variable features identified. Dimensionality reduction was performed using PCA, followed by clustering. Hepatocyte clusters were identified based on the expression of various known marker gene. Gene expression pattern in hepatocyte population were visualized using feature plot and violin plot.

O link analysis. Eighty patient plasma samples were profiled with the Olink Explore 1536 panel for quantitation of various proteins. Resulting protein concentrations calculated as Normalized Protein eXpression (NPX) values, calculated from Ct values and data pre-processing to minimize assay variation.

Cell culture

AML-12 mouse hepatocyte cell line. AML-12 (ATCC, Manassas, Virginia) was cultured in DMEM/F12 medium (Gibco, Billings, Montana) supplemented with 10% fetal bovine serum (FBS; HyClone, Logan, Utah) and 1% antibiotic–antifungal mixture (100 U/mL penicillin, 100 µg/mL streptomycin; Gibco). The cells were incubated at 37 °C with a CO_2 concentration of 5%. Passage

was carried out when the cell confluence reached approximately 70–80%. Upon reaching the logarithmic growth phase, AML-12 cells were seeded in a culture plate and incubated. Subsequently, the cells were treated with palbociclib for 5 days and recombinant TP for 2 days.

Huh7 human hepatoma cell line. Huh7 (ATCC) was cultured in DMEM supplemented with 10% FBS and 1% penicillin-streptomycin at 37 °C with a CO_2 concentration of 5%. Huh7 cells were seeded in a culture plate and treated with 200μM of oleate (Sigma-Aldrich, St. Louis, Missouri) and 100μM palmitic acid (Sigma-Aldrich, St. Louis, Missouri), OPA for 4 days to induce lipotoxicity. After that, recombinant TP or TP inhibitor (TPI-HCl) was treated into the cells for 2 days.

Primary mouse hepatocytes. Primary mouse hepatocytes were isolated from control and Smo deleted mice using a 2-step collagenase perfusion technique. Cell viability was determined by trypan blue exclusion and generally >95%. Cell purity was evaluated with an assessment of the relative percentage of hepatocytic-appearing cells using standard microscopy, and purity was generally > 96% hepatocytes. Cells were plated in DMEM F12 medium containing 5% FBS and 1% streptomycin and penicillin. After 4 hours, cells were washed and treated with 200μM of oleate (Sigma-Aldrich) and 100μM palmitic acid (Sigma-Aldrich) in DMEM F12 containing 2% FBS, 1% Penicillin/streptomycin, 1% L-glutamine (Gibco), 0.1% Insulin-transferrin-selenium (Gibco), and 40ng/ml Dexamethasone (Sigma-Aldrich). The cells were maintained for 4 days and fixed with 4% paraformaldehyde (Santa Cruz, Dallas, Texas) for following staining analysis.

siRNA-Smoothened (Smo) transfection

Huh7 cells (1.5×10^5) were seeded into 6 well plates and mixed with ON-TARGETplus siRNA SMARTPool human SMO (Horizon Discovery, Cambridge, UK; L-005726-00-0005; 20 nM) and of Lipofectamine™ 3000 Transfection Reagent (ThermoFisher Scientific, Waltham, MA; L3000001) for four days at 37°C and 5% CO_2 incubator.

Mitochondrial isolation

The Mitochondria Isolation Kit for Tissue (ThermoFisher Scientific) was used to isolate mitochondria from mouse total liver. Mitochondria were isolated as per standard protocols under sterile conditions at 4°C.

Immunoblot

Protein was extracted from total liver tissues or harvested cell pellets using RIPA buffer (Sigma-Aldrich) with Halt™ Protease & Phosphatase Single-Use Inhibitor Cocktail (ThermoFisher Scientific). Equal quantities of protein were loaded and subjected to SDS-PAGE gel electrophoresis using 4%-20% Criterion gels (BioRad, Hercules, CA). Subsequently, the proteins were transferred onto PVDF membranes and probed with the primary antibodies **(Supple Table 2)**. Blots were then incubated with HRP-conjugated secondary antibodies and visualized with Image Studio™ Lite Ver 5.2 (LI-COR Biosciences).

Immunohistochemistry

Liver tissue samples were fixed in formalin, embedded in paraffin, and sectioned. The sections were subjected to various staining techniques for histopathologic evaluation. Hematoxylin and eosin (H&E) staining was performed to assess overall liver histopathology. Sirius Red (Sigma-Aldrich, 365548) staining was utilized to evaluate liver fibrosis, following the manufacturer's instructions. Terminal deoxynucleotidyl transferase dUTP nick end labeling (TUNEL) staining was conducted to detect cell death using the In Situ Cell Death Detection Kit (Roche Diagnostics, Indianapolis, IN) as per the manufacturer's guidelines.

For immunohistochemistry, the slides were dewaxed, hydrated, and treated with 3% hydrogen peroxide for 10 minutes to block endogenous peroxidase activity. Antigen retrieval was performed by heating the slides in 10mmol/L sodium citrate buffer (pH 6.0) for 10 minutes. Slides were then blocked with Dako protein block solution (Agilent Technologies, Santa Clara, CA) for 1 hour and incubated overnight at 4°C with specific primary antibodies **(Supple Table 2)**. Polymer-

horseradish peroxidase secondary antibodies were applied for 1 hour at room temperature, followed by detection using the Dako 3,3'-Diaminobenzidine Substrate Chromogen System. To detect liver DNA damage, slides were incubated with anti-gamma H2A.X and anti-8-Hydroxy-2'-deoxyguanosine primary antibodies, and then with Alexa Fluor™ 488 and Alexa Fluor™ 594 secondary antibodies (ThermoFisher Scientific) for fluorescence detection. Images were acquired and processed using Leica Microsystems (Leica, Wetzlar, Germany).

Frozen liver tissue samples were also used. Sections were cut at a thickness of 20μm, fixed with 10% formalin, and stained with Oil Red O (Sigma-Aldrich, O0625) for 15 minutes to visualize lipid accumulation. Cellular senescence in the liver was evaluated by SA-β-gal staining using a commercially available kit (Cell Signaling, 9860), following the manufacturer's instructions. Results were examined using light microscopy.

Immunoprecipitation

Mouse primary hepatocytes were scraped off the culture dish with 1 ml of cold RIPA buffer (Sigma-Aldrich) containing Halt™ Protease & Phosphatase Single-Use Inhibitor Cocktail (ThermoFisher Scientific). The cell lysate was centrifuged at 13,000g for 15 minutes at 4°C. The supernatant was harvested, and its protein concentration was measured with a BCA protein assay kit. For immunoprecipitation, 300 μg of proteins were incubated with a Protein A/G PLUS-Agarose (Santa Cruz Biotechnology, Dallas, Texas; sc-2003) for 1 hour at 4°C. The pre-mixture of protein and protein A/G agarose beads were incubated with specific primary antibodies overnight at 4°C with constant rotation **(Supple Table 2)**. After centrifugation at 1000 rpm for 1 minute at 4°C, the supernatant was discarded, and the agarose-bead pellets were washed with PBS. After the final wash, the agarose-bead pellets were re-suspended in a final volume of 20 μl with sample buffer (50 mM TRIS–HCl pH 6.8, 2 % SDS, 10 % glycerol, 1 % β-mercaptoethanol, 12.5 mM EDTA, 0.02 % bromophenol blue) and boiled at 95°C for 10 minutes. The samples were loaded immediately onto an SDS-PAGE gel electrophoresis using 4%-20% Criterion gels and

immunoblots were performed as described above.

Cytokine array analysis

The Proteome Profiler Mouse XL Cytokine Array Kit (R&D systems, Minneapolis, MN; ARY028), a multiplex antibody array system including 111 cytokines, chemokines, and growth factors of mouse antibodies, was used to assess protein levels of secretomes in serum and culture supernatants. The assay allows the simultaneous detection of 111 proteins in the plate using a single biological sample in an unbiased manner; as the method relies on chemiluminescent detection of antibody bound proteins, values are given in relative units instead of absolute concentrations. Image J was used to quantify signal of each spot.

Enzyme-linked immunosorbent (ELISA) assay

The concentration of malondialdehyde (MDA), 4-hydroxynonenal (4-HNE), thymidine phosphorylase (TP), glucose, and insulin in total liver, serum of mice and culture supernatant were analyzed by ELISA. Their concentrations were measured using mouse MDA (MyBioSource, San Diego, CA; MBS741034), 4-HNE (MyBioSource, MBS7607148), TP (Bioss Inc., Woburn, MA; BSKM61675), glucose (abcam, ab65333), and insulin (Crystal Chem, Elk Grove Village, IL; 90080) ELISA kits in strict accordance with the manufacturer's instructions and detected using a microplate reader (Tecan, Mannedorf, Switzerland) at 450nm.

Seahorse extracellular flux (XF) assay

Cellular oxygen consumption rate (OCR) was monitored in real time using the Seahorse Bioscience Extracellular Flux Analyzer (XF96e, Agilent, Santa Clara, CA). Assays were performed in 9-min cycles of mix (3 min), wait (3 min), and measure (3 min) as per the manufacturer's recommendations. For mitochondrial stress test assays, cells were sequentially treated as indicated with oligomycin (1μM), carbonyl cyanide 4-(trifluoromethoxy)phenylhydrazone (FCCP; 1μM), and a combination of antimycin A (0.5μM) and rotenone (0.5μM). Cellular metabolism

readouts, such as basal respiration, glycolysis, and ATP production, were determined using the Agilent Seahorse Wave software.

Supplemental Table 1: Patient characteristics.

	Control (n = 69)	MASLD (n = 299)	*p* value
**Gender (% female)**	81	61	0.013
**Age (year)**	47.4 ± 12.6	50.5 ± 12.8	0.07
**BMI (kg/m^2)**	37.4 ± 8.2	36.8 ± 8.1	0.58
**Diabetes**	17.4	47.8	< 0.0001
**Hyperlipidemia**	46.4	34.4	0.07
**Hypertension**	43.5	32.4	0.17
**Fibrosis stage**			
**0**	100	10	< 0.0001
**1**	-	22.4	
**2**	-	35.8	
**3**	-	27.1	
**4**	-	4.7	
**NAS score**			
**≤3**	-	1.7	
**4**	-	35.5	
**5**	-	36.5	
**6**	-	18.7	
**≥7**	-	7.7	

Values are % or mean ± SD.

Supplemental Table 2: Primary antibody used for immuno-blot, histochemistry, and precipitation

Antibody	Host Animal	Dilution for Immunoblot	Dilution for Immunohistochemistry	Dilution for Immunoprecipitation	Distributor	Cat. Num.
**AIF**	Rabbit	1:1,000	-	-	Cell Signaling	5318S
**ASMA**	Rabbit	-	1:200	-	abcam	ab32575
**β-tubulin**	Rabbit	1:3,000	-	-	abcam	ab6046
**CDKN2A/p16INK4a (p16)**	Rabbit	1:500	-	-	abcam	ab211542
**Collagen 1**	Rabbit	1:1,000	-	-	abcam	ab34710
**COX 4**	Rabbit	1:1,000	-	-	Cell Signaling	4850S
**Cytochrome c**	Rabbit	1:1,000	-	-	Cell Signaling	4280S
**Desmin**	Rabbit	1:1,000	-	-	abcam	ab32362
**F480**	Rabbit	-	1:200	-	Cell Signaling	70076S
**gamma H2A.X**	Rabbit	-	1:200	-	abcam	ab81299
**GAPDH**	Rabbit	1:3,000	-	-	Cell Signaling	3683S
**GDF15**	Rabbit	-	1:200		Bioss	BS-3818R
**Gli2**	Rabbit	-	1:200	-	Aviva systems	OARA02754
**SDHA**	Rabbit	1:1,000	-	-	Cell Signaling	11998S
**Heme Oxygenase 1**	Rabbit	1:1,000	1:400	1:200	abcam	ab189491
**HSP60**	Rabbit	1:1,000	-	-	Cell Signaling	12165S
**Nrf2**	Rabbit	1:1,000	-	1:100	Cell Signaling	12721S
**OXPHOS**	Mouse	1:2,000	-	-	abcam	ab110413
**PGC1a**	Rabbit	-	1:200	-	Novus Biologicals	NBP1-04676
**PHB1**	Rabbit	1:1,000	-	-	Cell Signaling	2426S
**Phospho-Nrf2 (Ser40)**	Rabbit	-	1:200	-	ThermoFisher Scientific	PA5-67520
**Pyruvate Dehydrogenase (PDH)**	Rabbit	1:1,000	-	-	Cell Signaling	3205S
**p21**	Rabbit	1:1,000	1:200	-	abcam	ab188224
**Smac/Diablo**	Rabbit	1:1,000	-	-	Cell Signaling	15108S
**Smo**	Rabbit	1:1,000	-	1:100	MyBioSource	MBS129037
	Rabbit	1:1,000	-	-	Cell Signaling	92981S
**SOD**	Mouse	1:1,000	-	-	Cell Signaling	4266S
**Thymidine phosphorylase (TP)**	Rabbit	1:1,000	1:200	-	Cell Signaling	4307S
	Rabbit	1:1,000	1:200	1:100	MyBioSource	MBS8247716
**VDAC**	Rabbit	1:1,000	-	-	Cell Signaling	4661S
**Vimentin**	Rabbit	1:1,000	-	-	abcam	ab92547
**8OHDG**	Mouse	-	1:200	-	Novus Biologicals	NB600-1508

Supplemental Data

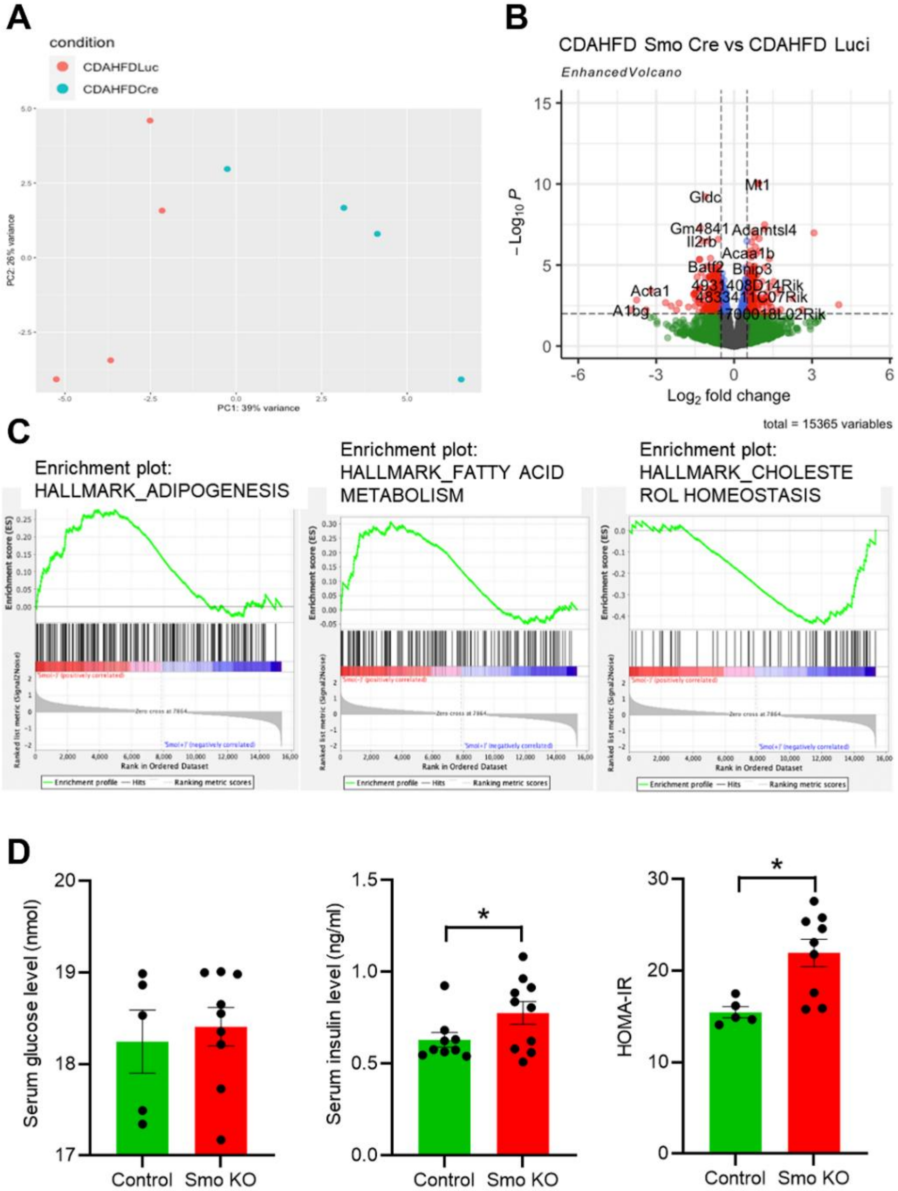

Supplemental Figure 1. Gene signatures and metabolic parameters of Smo KO mice fed CDA-HFD. (A) PCA reveals that control and Smo KO have difference with the liver genes. **(B)** DEGs shows different gene signatures between control and Smo KO liver. **(C)** GSEA demonstrated that control and Smo KO have difference with adipogenesis, fatty acid metabolism, and cholesterol homeostasis *(n=3* mice per group). **(D)** Serum levels of insulin, glucose and HOMA-IR in Smo KO and control mice demonstrate insulin resistance in mice with Smo-depleted hepatocytes. P values were calculated using one-way ANOVA. Data are graphed as mean ± SEM. * $p < 0.05$.

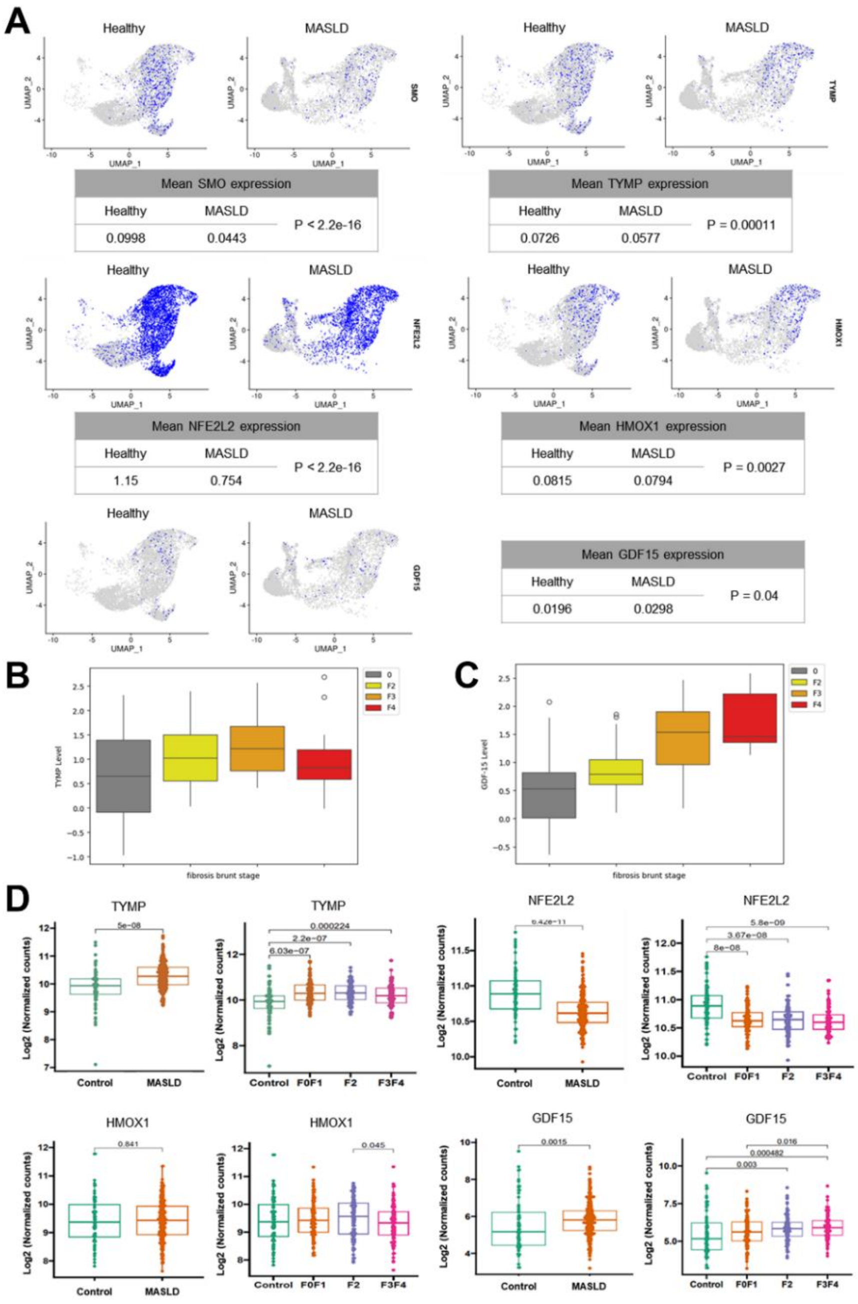

Supplemental Figure 2. Smo and antioxidant gene expressions in human livers with MASLD. (A) Single nuclei RNA seq data showing expression of SMO, TYMP, NFE2L2, HMOX1 and GDF15 in hepatocyte from Healthy and MASLD livers *(n=2* subjects per groups). Plasma protein level of TYMP **(B)** and GDF15 **(C)** across different fibrosis stage *(n=20* controls; *n=20* MASLD patients with F2 fibrosis; *n=20* MASLD patients with F3 fibrosis; *n=12* MASLD subjects with F4 fibrosis). **(D)** Bulk RNA seq data showing expression of TYMP, NFE2L2, HMOX1 and GDF15 in control and MASLD liver, and across different fibrosis stage of MASLD (*n=69 controls*; *n=299* MASLD patients; fibrosis F0F1, *n=97*; F2, *n=107*; F3F4, *n=95)*. P values were calculated using Wilcoxon Rank Sum test.

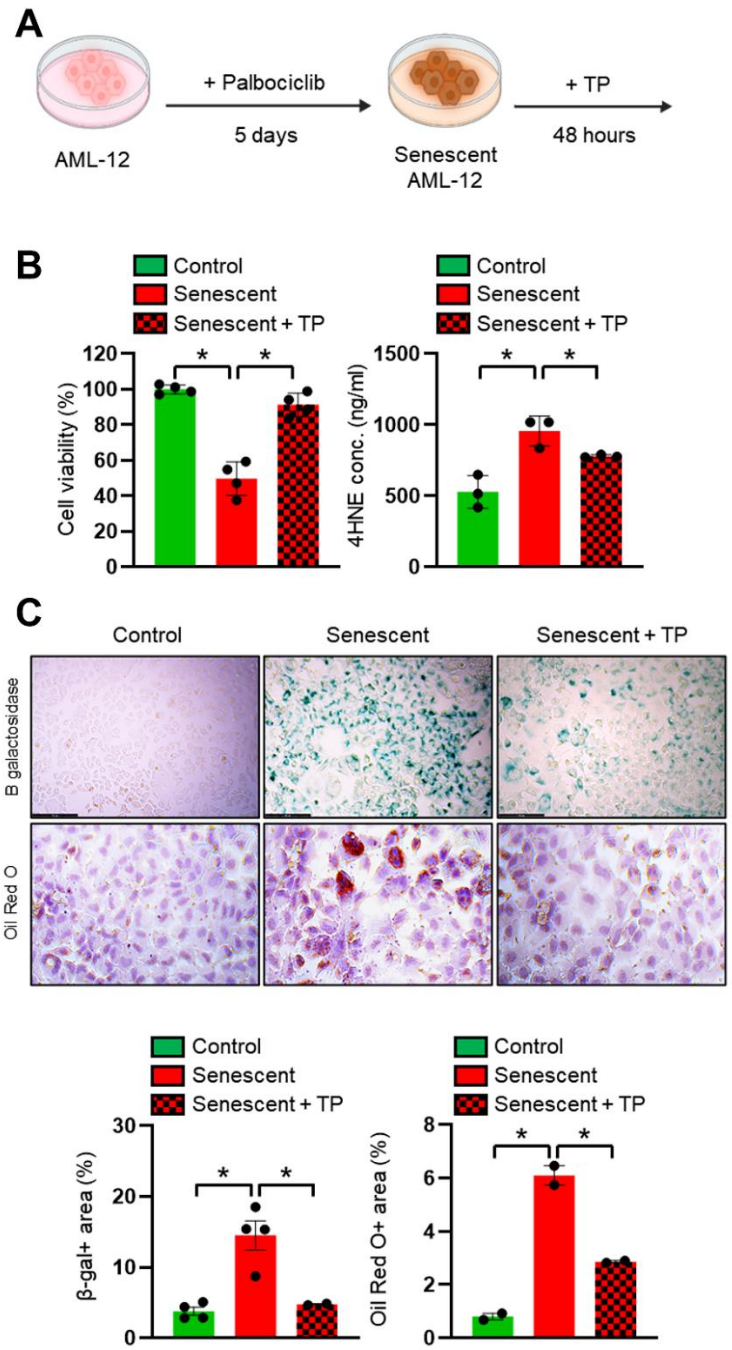

Supplemental Figure 3. TP attenuates senescence and lipotoxicity in senescent hepatocytes. (A) Experimental scheme. **(B)** Cell viability and 4HNE levels in palbociclib treated AML-12 cells by CCK-8 and ELISA assay, respectively **(C)** beta-galactosidase and Oil Red O staining and corresponding morphometric quantification. P values were calculated using one-way ANOVA. Data from triplicate experiments are graphed as mean ± SEM. * $p < 0.05$.

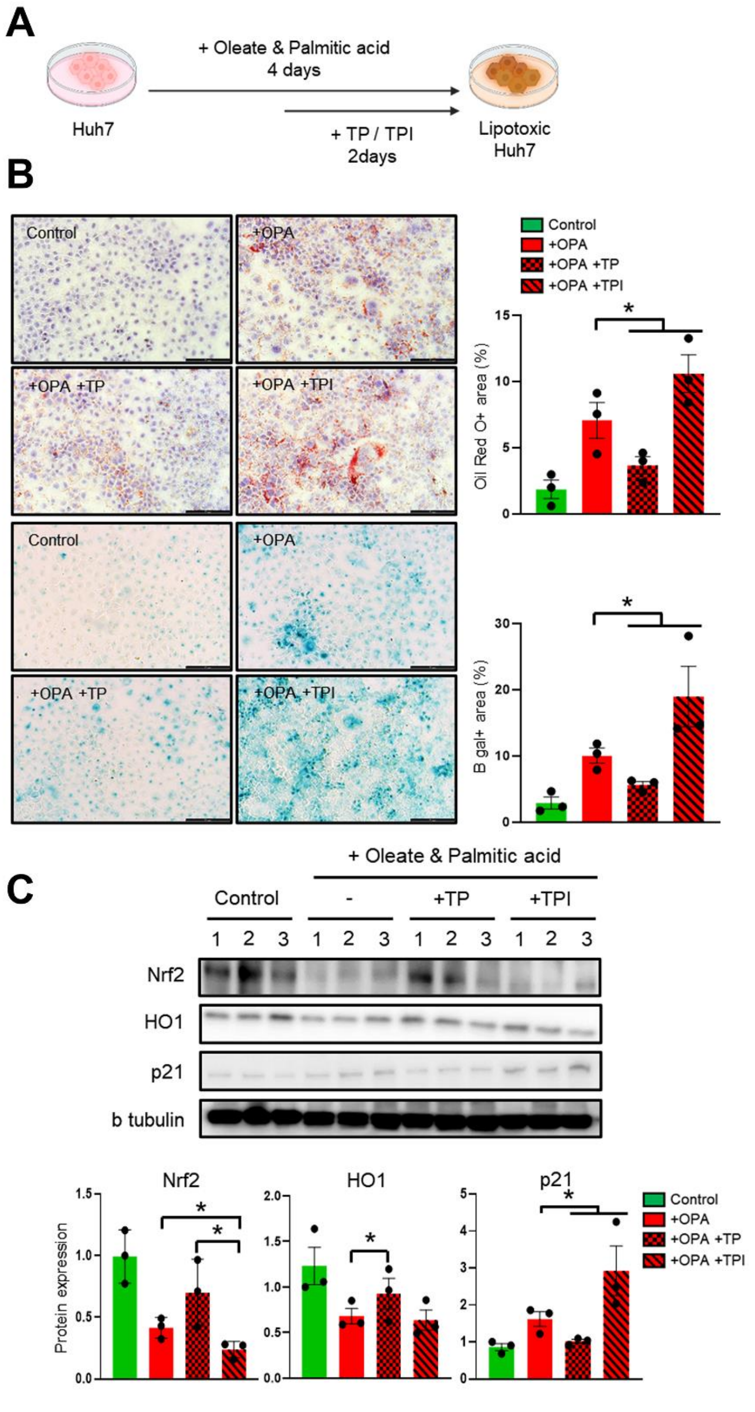

Supplemental Figure 4. TP enhances antioxidant defense and attenuates lipotoxicity and senescence during lipotoxic stress. (A) Experimental scheme. **(B)** Oil Red O and B gal staining and corresponding morphometric quantification. **(C)** Protein expressions of Nrf2, HO1, and p21 by immunoblot in lipotoxic Huh7 cells. Data from triplicate experiments are graphed. P values were calculated using one-way ANOVA. Data are graphed as mean ± SEM. * $p < 0.05$.

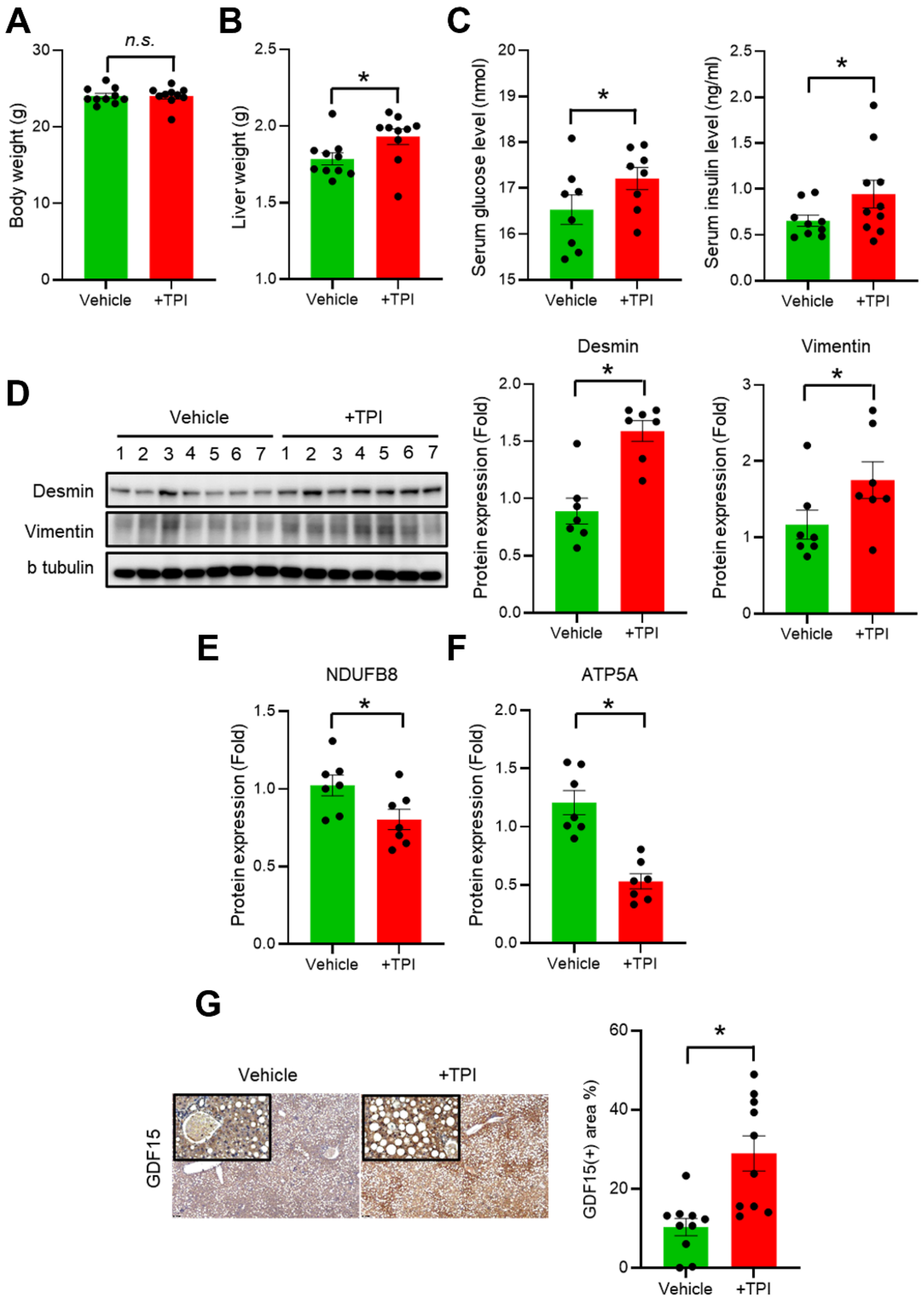

Supplemental Figure 5. Inhibiting TP induces hepatomegaly and liver fibrosis in CDA-HFD fed wild type mice. (A) Body and **(B)** Liver weight in TPI treated versus vehicle treated mice. **(C)** Serum glucose, insulin, and HOMA-IR in TPI-treated versus vehicle treated mice *(n=10 mice* per group). **(D)** Protein expressions of fibrotic markers, Desmin and Vimentin, detected in TPI treated mice total liver by immunoblot. The beta tubulin blot is also shown in Figure 5F. Mitochondrial protein expressions of **(E)** NDUFB8 and **(F)** ATP5A based on immunoblot *(n=7* mice per group). **(G)** representative staining of GDF15 and quantification of positively stained area in vehicle or TPI treated mice liver fed with CDA-HFD for 6 weeks *(n=10* mice per group). P values were calculated using one-way ANOVA. Data are graphed as mean ± SEM. * $p < 0.05$.
