# Supplementary material for: The senescence-associated secretome of Hedgehog-deficient hepatocytes drives MASLD progression
Source: J Clin Invest. 2024 Aug 27;134(19):e180310. doi: 10.1172/JCI180310 (PMC11444248; doi:10.1172/JCI180310)

Original images for Blots

Full western blot gel panels

Figure 1D

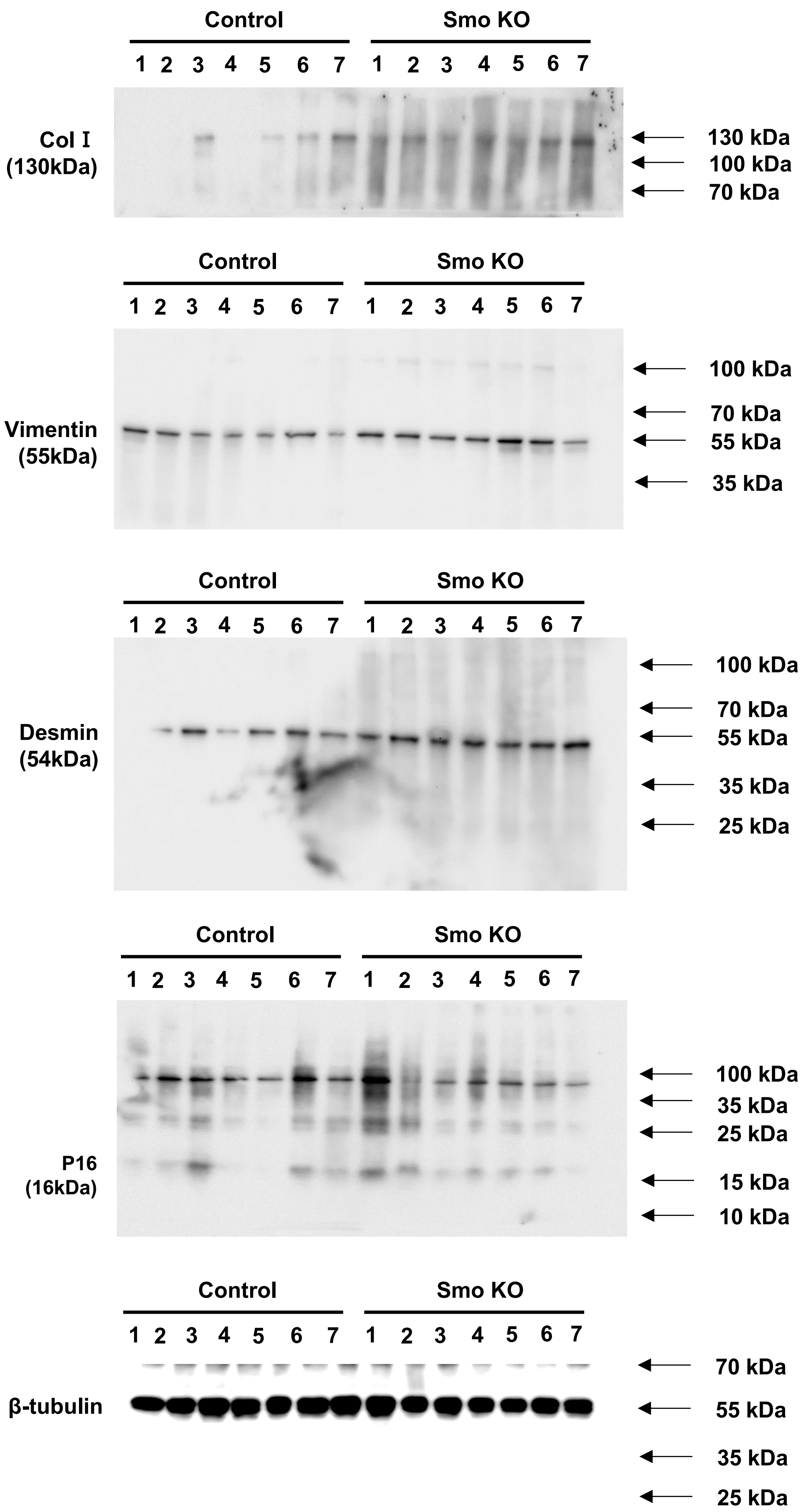

Control
Smo KO
1 2 3 4 5 6 7 1 2 3 4 5 6 7
Col I (130kDa)
130 kDa
100 kDa
70 kDa
Control
Smo KO
1 2 3 4 5 6 7 1 2 3 4 5 6 7
Vimentin (55kDa)
100 kDa
70 kDa
55 kDa
35 kDa
Control
Smo KO
1 2 3 4 5 6 7 1 2 3 4 5 6 7
Desmin (54kDa)
100 kDa
70 kDa
55 kDa
35 kDa
25 kDa
Control
Smo KO
1 2 3 4 5 6 7 1 2 3 4 5 6 7
P16 (16kDa)
100 kDa
35 kDa
25 kDa
15 kDa
10 kDa
Control
Smo KO
1 2 3 4 5 6 7 1 2 3 4 5 6 7
β-tubulin
70 kDa
55 kDa
35 kDa
25 kDa

Figure 2C

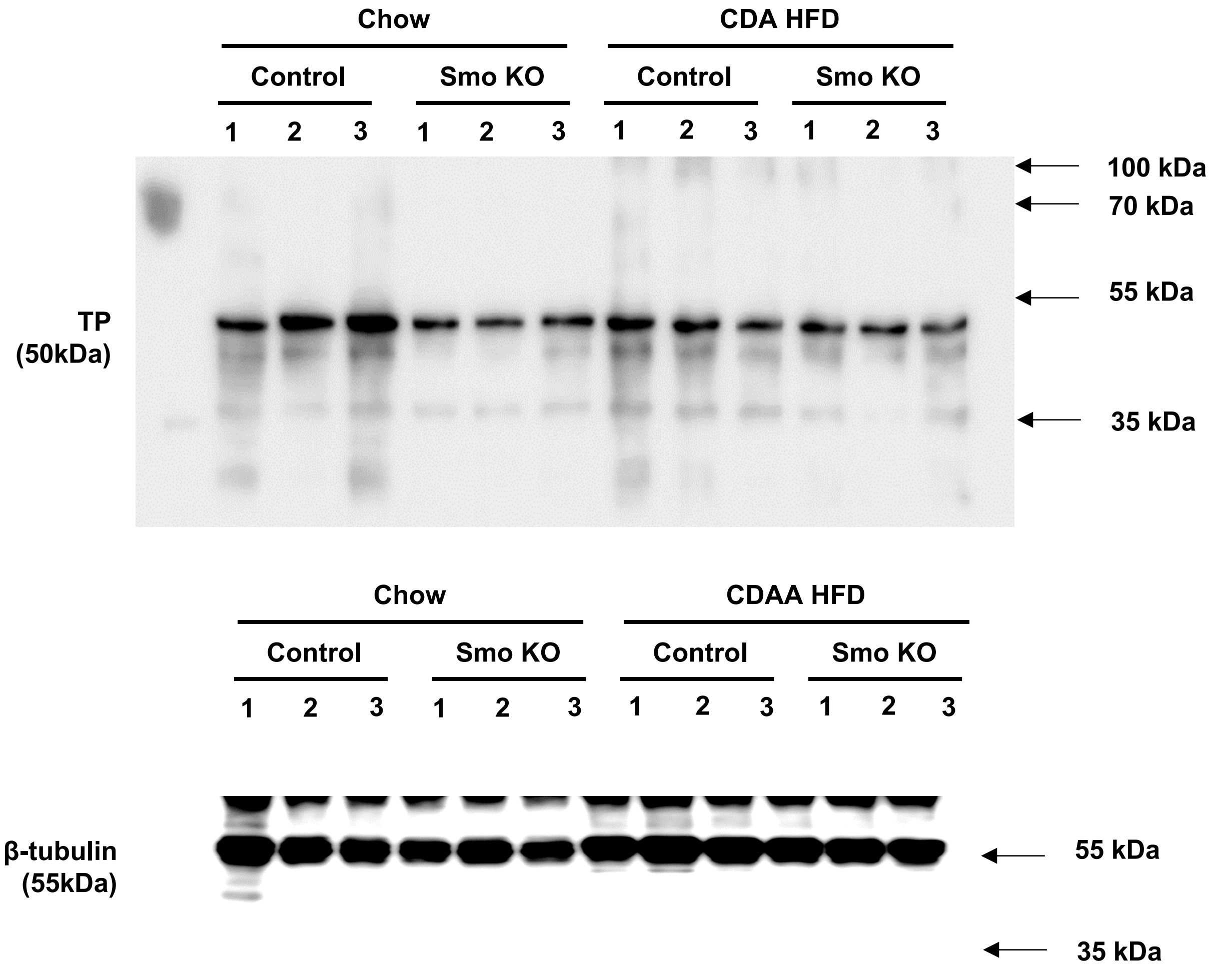

Chow
CDA HFD
Control
Smo KO
Control
Smo KO
1 2 3 1 2 3 1 2 3 1 2 3
100 kDa
70 kDa
55 kDa
35 kDa
TP
(50kDa)
Chow
CDAA HFD
Control
Smo KO
Control
Smo KO
1 2 3 1 2 3 1 2 3 1 2 3
β-tubulin
(55kDa)
55 kDa
35 kDa

Figure 3B

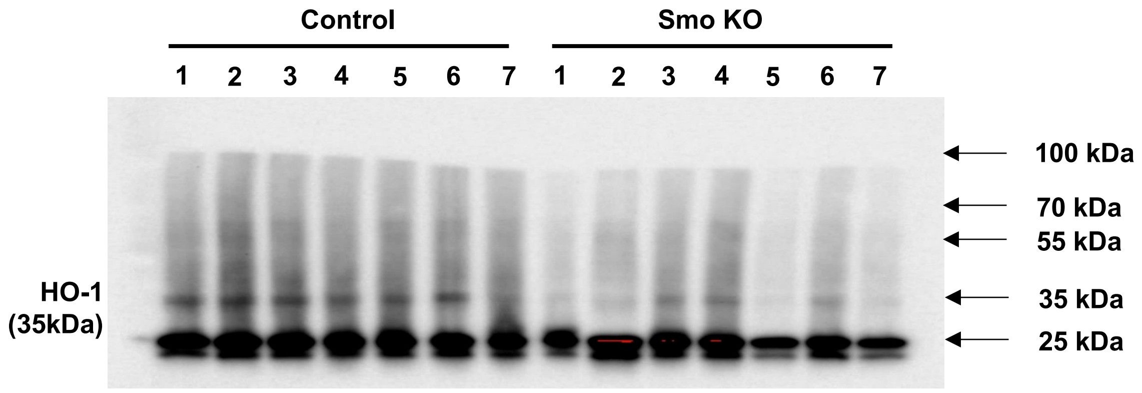
Control
Smo KO
1 2 3 4 5 6 7 1 2 3 4 5 6 7
HO-1
(35kDa)
100 kDa
70 kDa
55 kDa
35 kDa
25 kDa

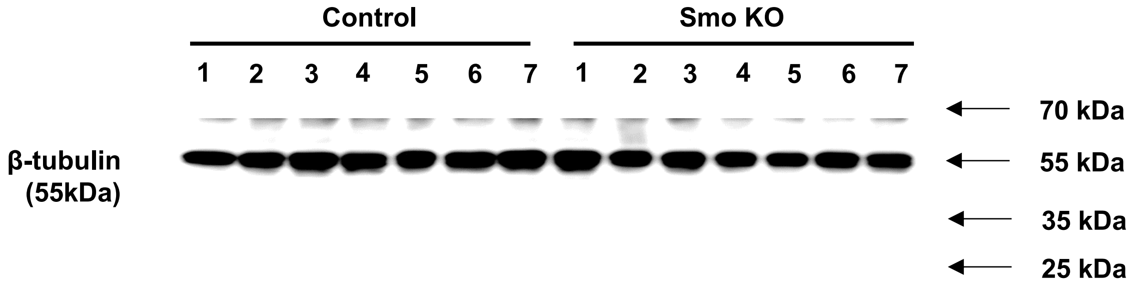
Control
Smo KO
1 2 3 4 5 6 7 1 2 3 4 5 6 7
β-tubulin
(55kDa)
70 kDa
55 kDa
35 kDa
25 kDa

Figure 3C

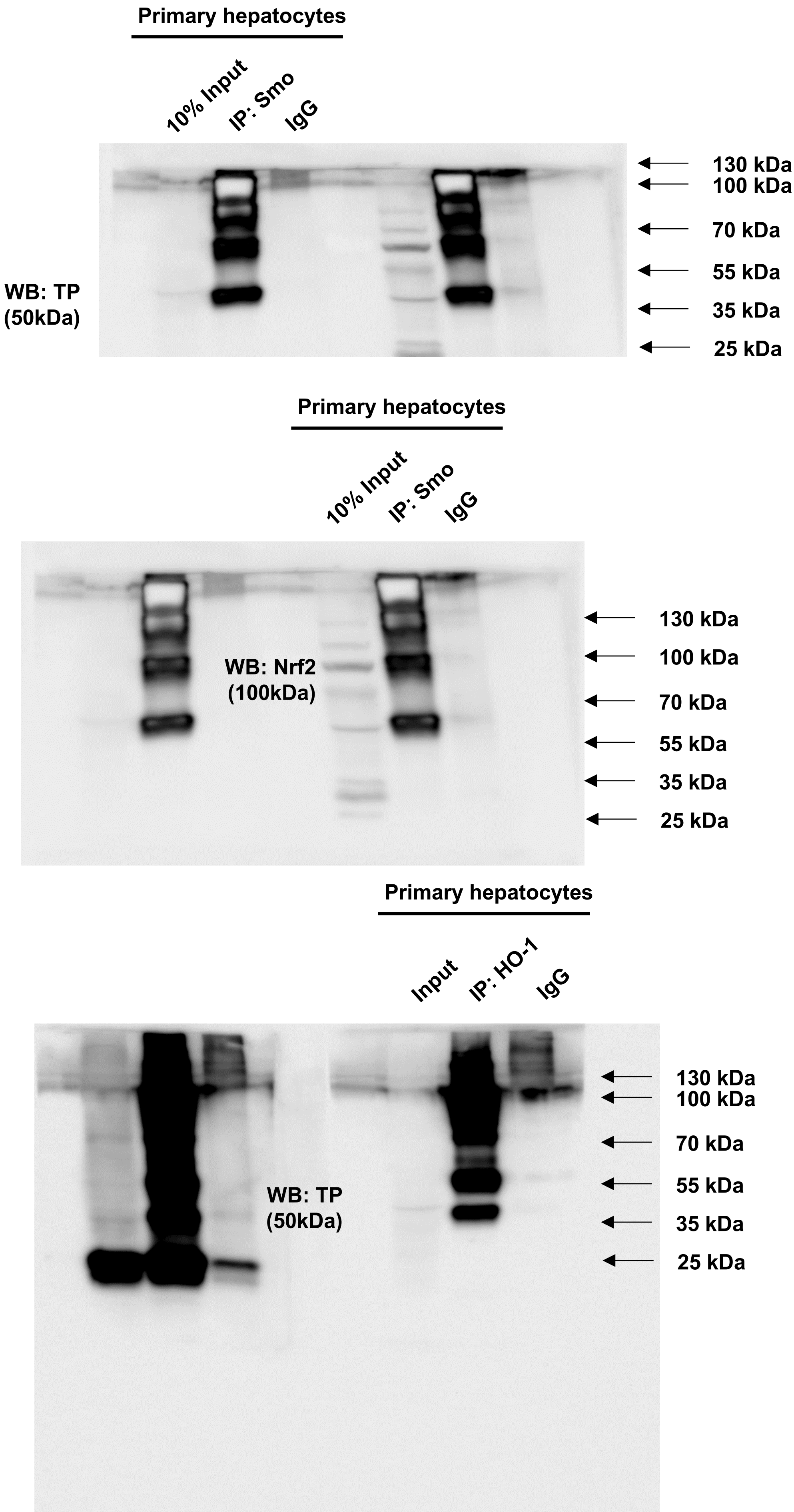

Primary hepatocytes
10% Input
IP: Smo
IgG
WB: TP
(50kDa)
130 kDa
100 kDa
70 kDa
55 kDa
35 kDa
25 kDa
Primary hepatocytes
10% Input
IP: Smo
IgG
WB: Nrf2
(100kDa)
130 kDa
100 kDa
70 kDa
55 kDa
35 kDa
25 kDa
Primary hepatocytes
Input
IP: HO-1
IgG
WB: TP
(50kDa)
130 kDa
100 kDa
70 kDa
55 kDa
35 kDa
25 kDa

Figure 3D

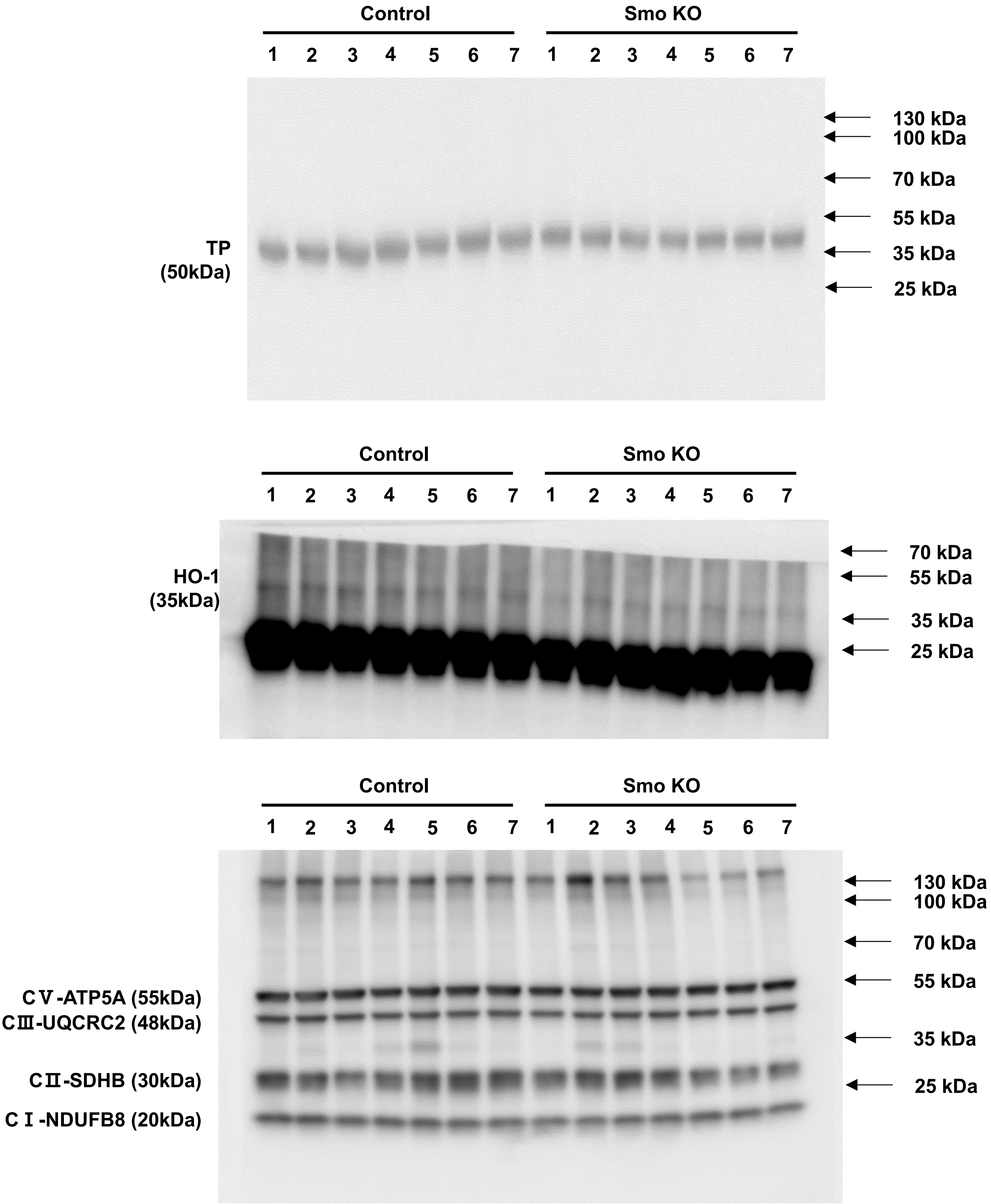

Control
Smo KO
1 2 3 4 5 6 7 1 2 3 4 5 6 7
TP
(50kDa)
130 kDa
100 kDa
70 kDa
55 kDa
35 kDa
25 kDa
Control
Smo KO
1 2 3 4 5 6 7 1 2 3 4 5 6 7
HO-1
(35kDa)
70 kDa
55 kDa
35 kDa
25 kDa
Control
Smo KO
1 2 3 4 5 6 7 1 2 3 4 5 6 7
CⅤ-ATP5A (55kDa)
CⅢ-UQCRC2 (48kDa)
CⅡ-SDHB (30kDa)
CⅠ-NDUFB8 (20kDa)
130 kDa
100 kDa
70 kDa
55 kDa
35 kDa
25 kDa

Figure 3D (Continued)

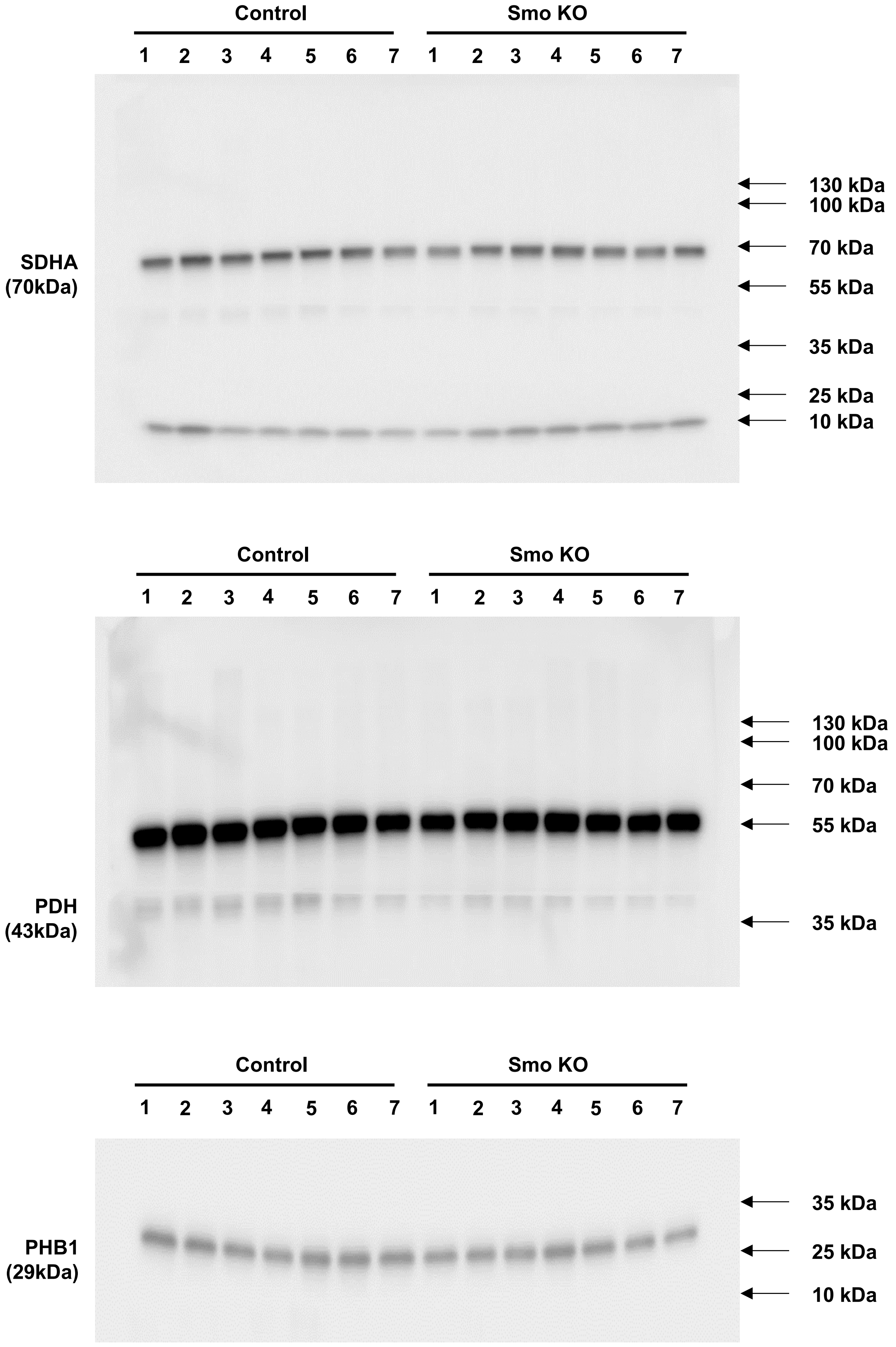

Figure 3D (Continued)

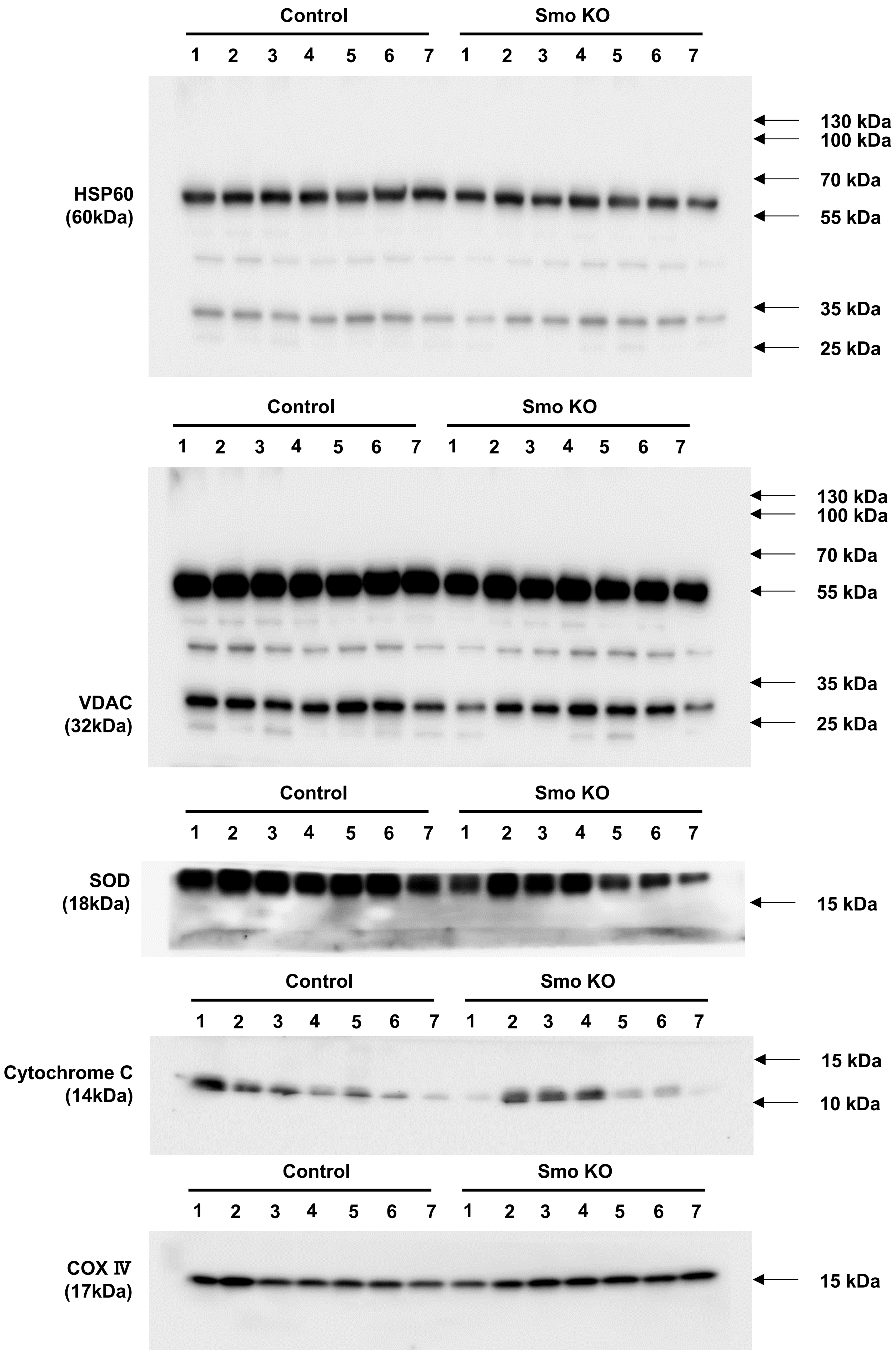

Figure 3F

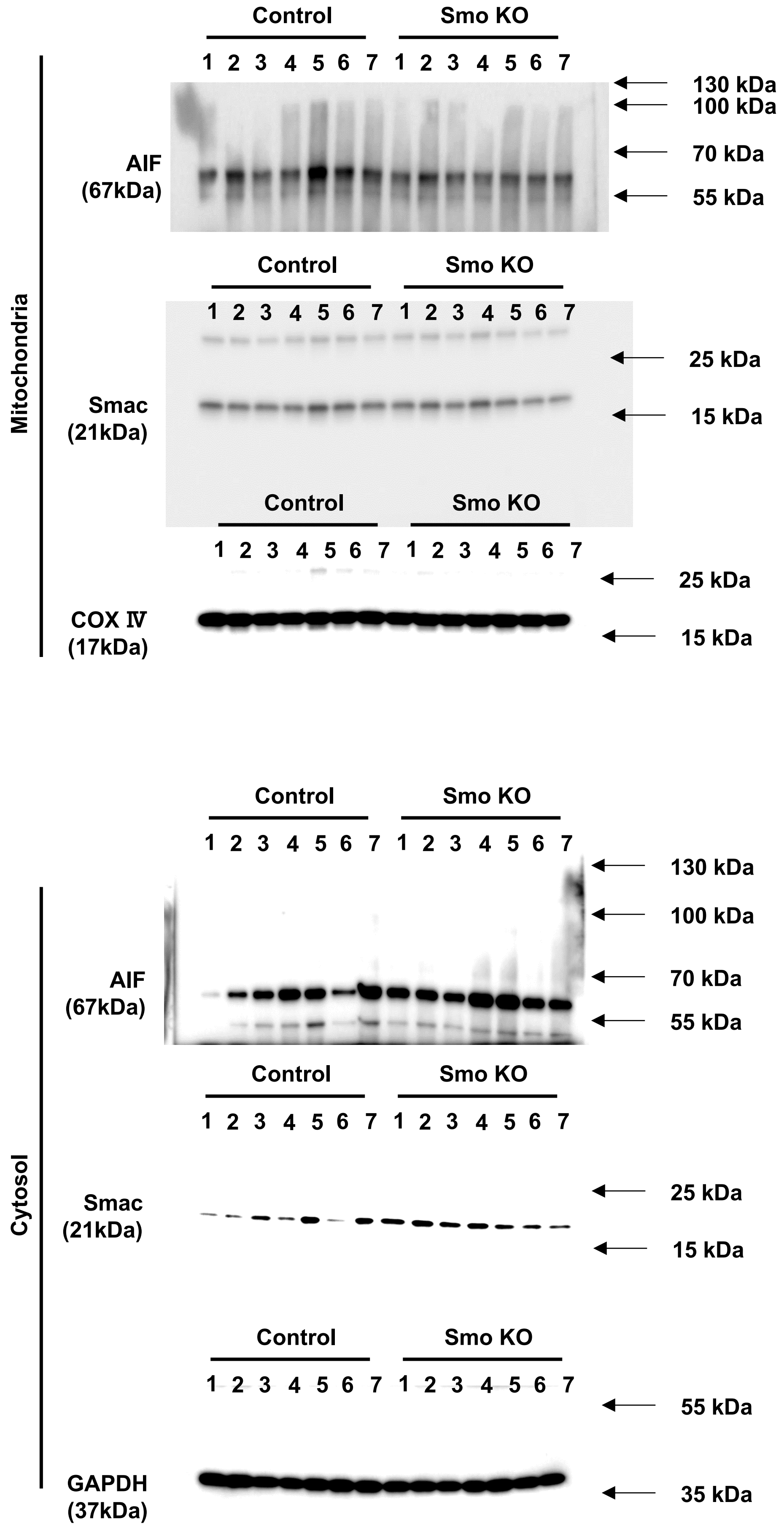

Mitochondria
Control
Smo KO
1 2 3 4 5 6 7 1 2 3 4 5 6 7
AIF
(67kDa)
130 kDa
100 kDa
70 kDa
55 kDa
Smac
(21kDa)
25 kDa
15 kDa
COX Ⅳ
(17kDa)
Cytosol
GAPDH
(37kDa)
35 kDa

Figure 4H

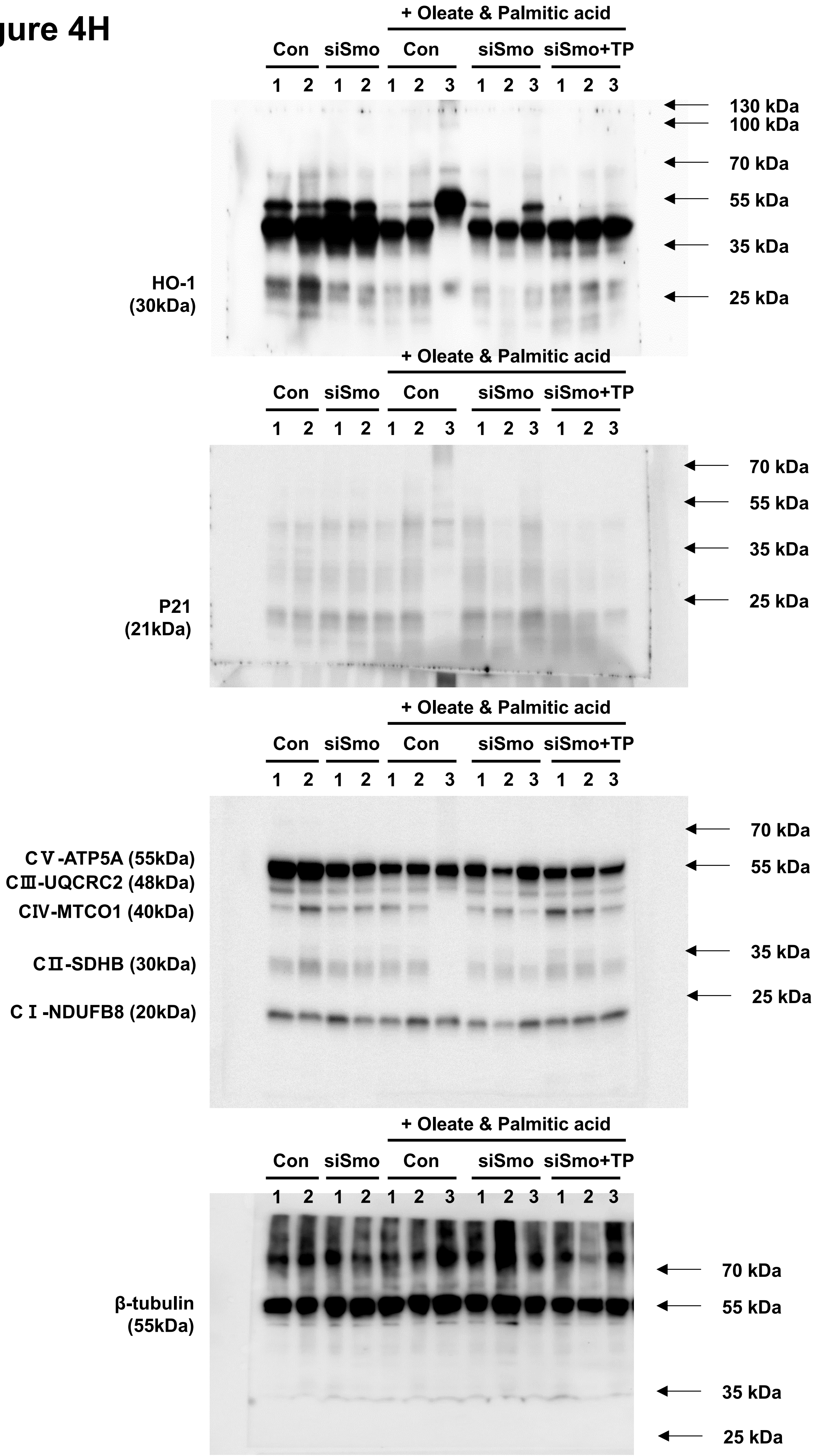
+ Oleate & Palmitic acid
Con
siSmo
Con
siSmo
siSmo+TP
1 2 1 2 1 2 3 1 2 3 1 2 3
130 kDa
100 kDa
70 kDa
55 kDa
35 kDa
25 kDa
HO-1
(30kDa)
P21
(21kDa)
CⅤ-ATP5A (55kDa)
CⅢ-UQCRC2 (48kDa)
CIV-MTCO1 (40kDa)
CⅡ-SDHB (30kDa)
CⅠ-NDUFB8 (20kDa)
β-tubulin
(55kDa)

Figure 5F

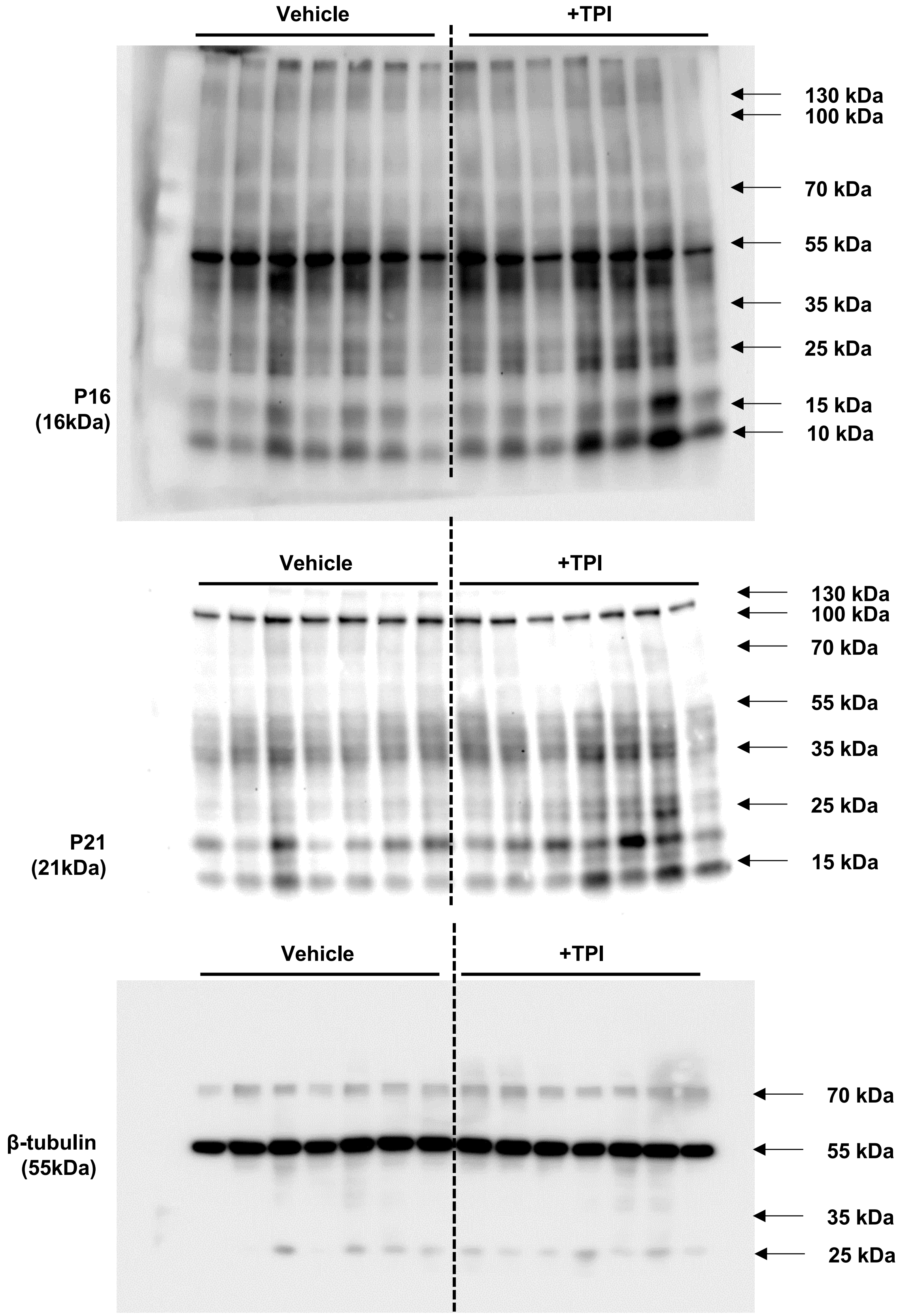

This is second appearance.

Figure 5H

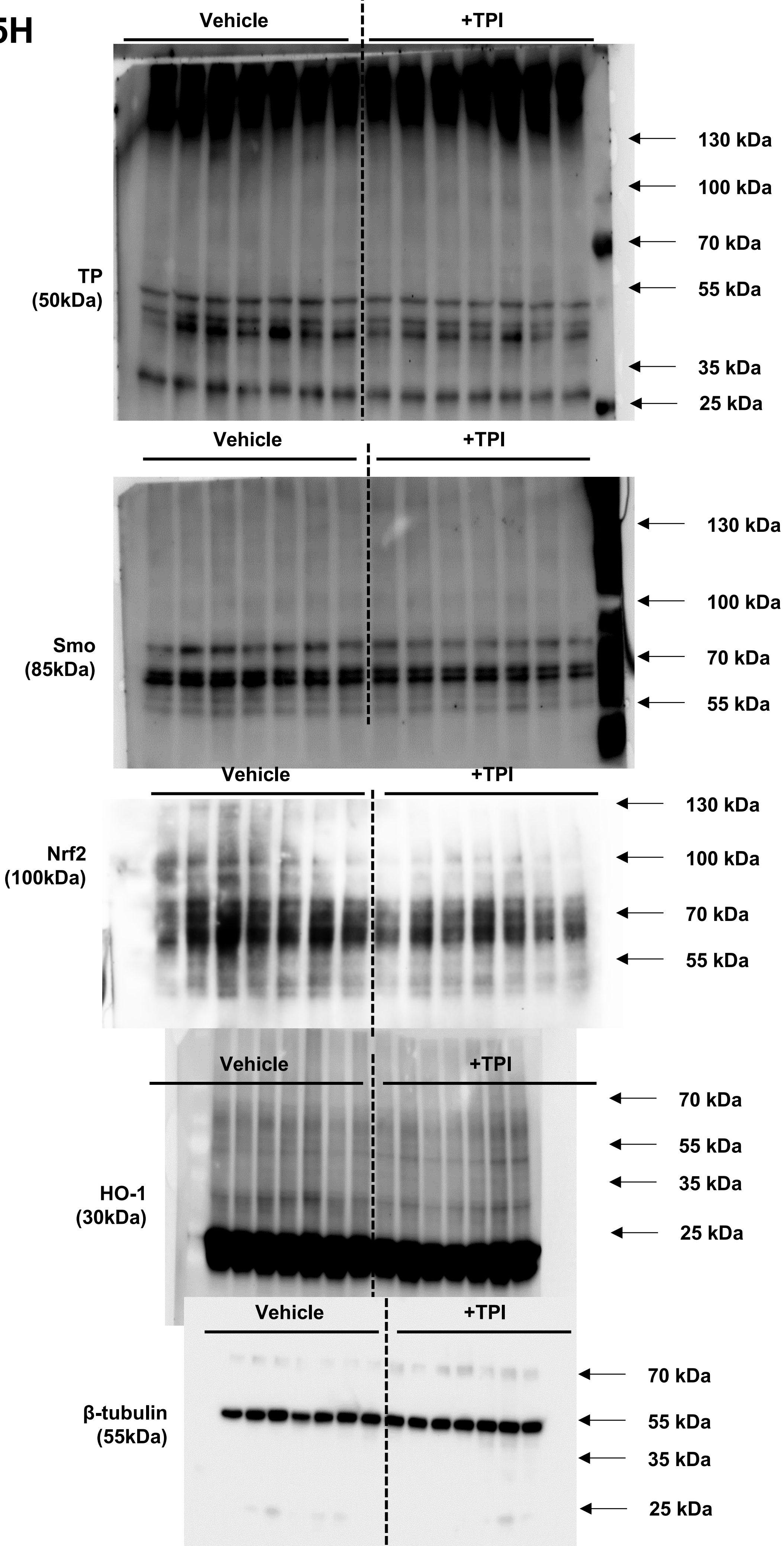
Vehicle
+TPI
TP
(50kDa)
130 kDa
100 kDa
70 kDa
55 kDa
35 kDa
25 kDa
Vehicle
+TPI
Smo
(85kDa)
130 kDa
100 kDa
70 kDa
55 kDa
Vehicle
+TPI
Nrf2
(100kDa)
130 kDa
100 kDa
70 kDa
55 kDa
Vehicle
+TPI
HO-1
(30kDa)
70 kDa
55 kDa
35 kDa
25 kDa
Vehicle
+TPI
β-tubulin
(55kDa)
70 kDa
55 kDa
35 kDa
25 kDa

Figure 5I

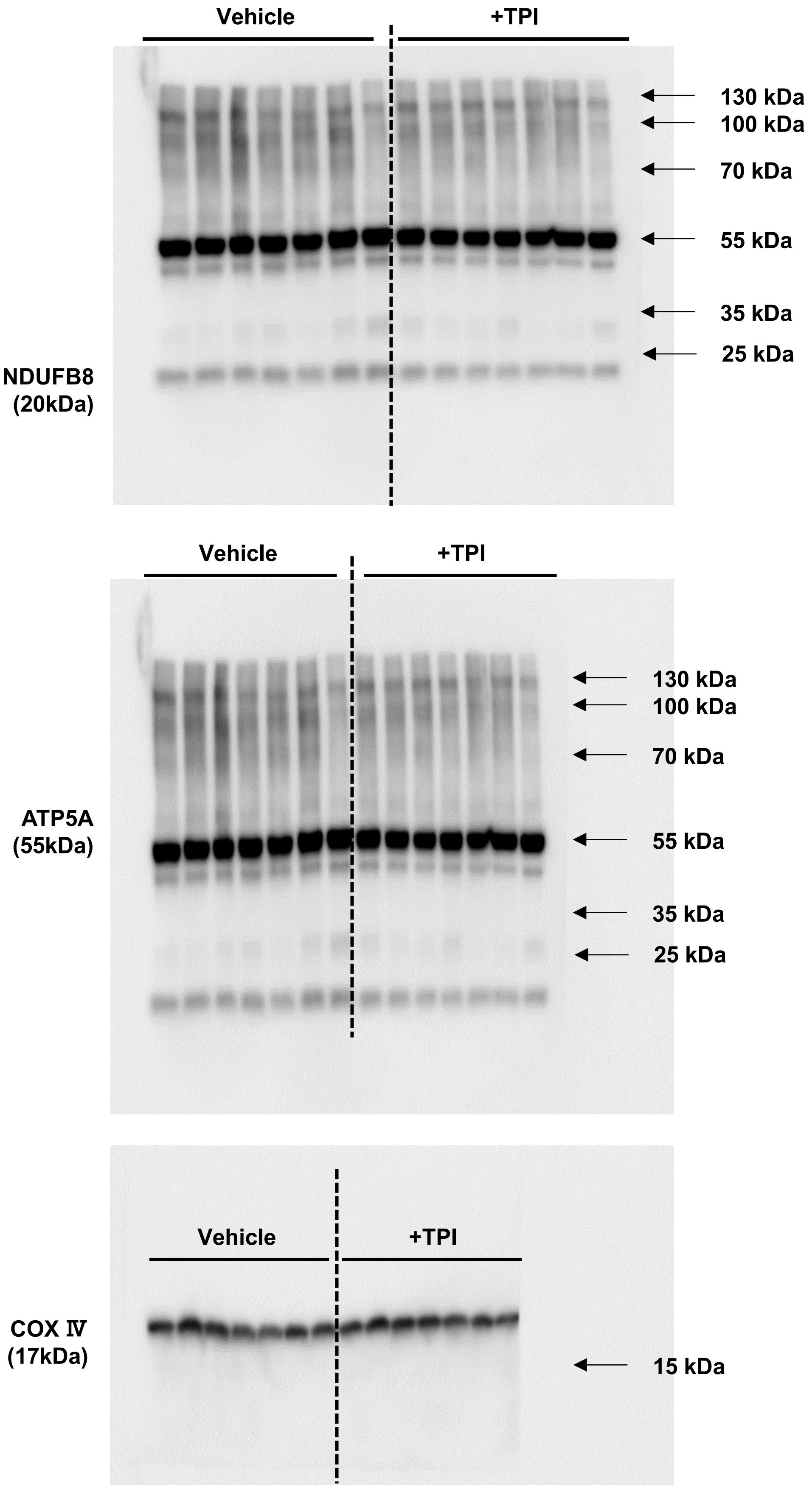
Vehicle
+TPI
130 kDa
100 kDa
70 kDa
55 kDa
35 kDa
25 kDa
NDUFB8
(20kDa)
Vehicle
+TPI
130 kDa
100 kDa
70 kDa
55 kDa
35 kDa
25 kDa
ATP5A
(55kDa)
Vehicle
+TPI
COX Ⅳ
(17kDa)
15 kDa

Supplemental Figure 4

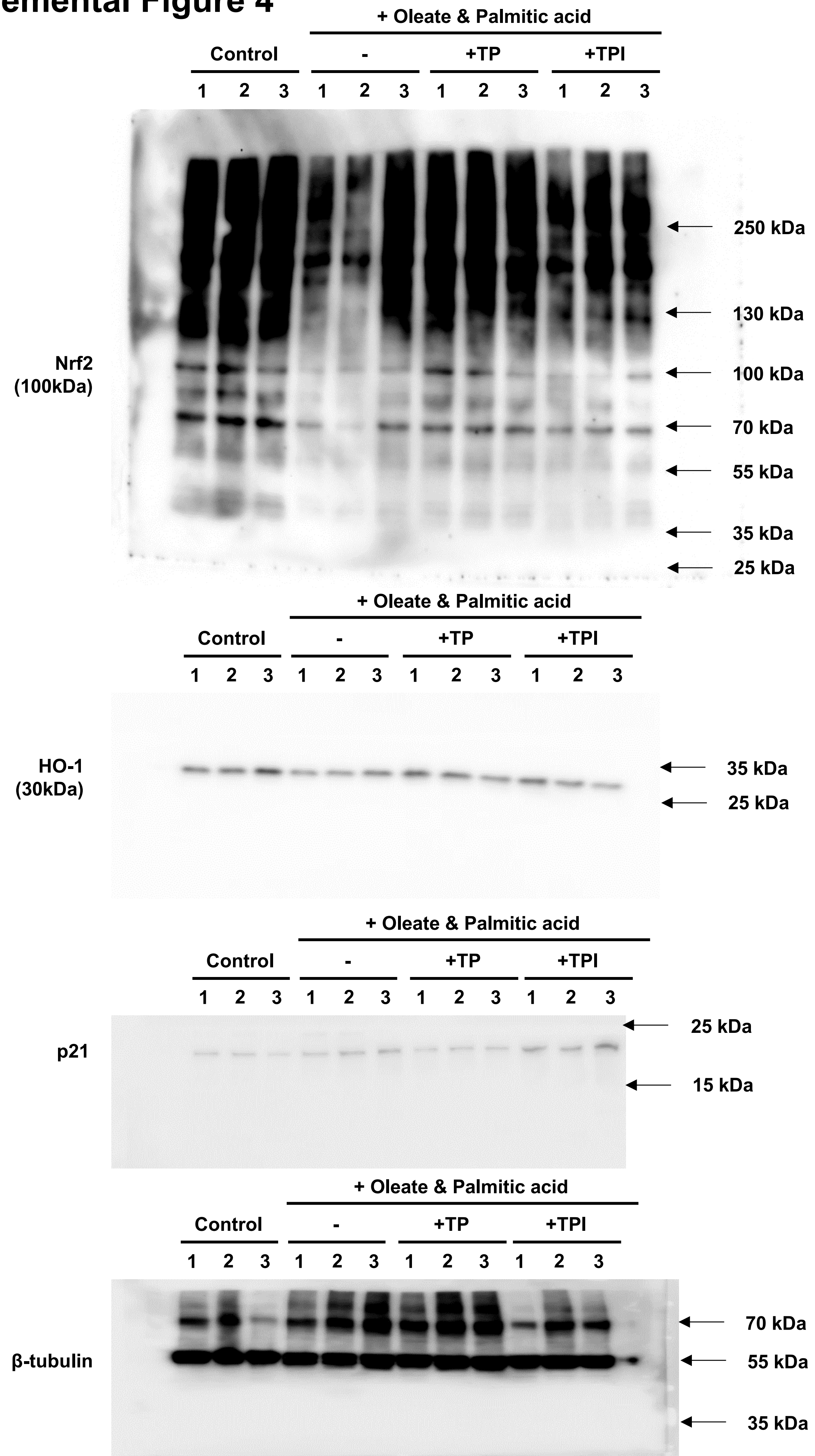

Supplemental Figure 5

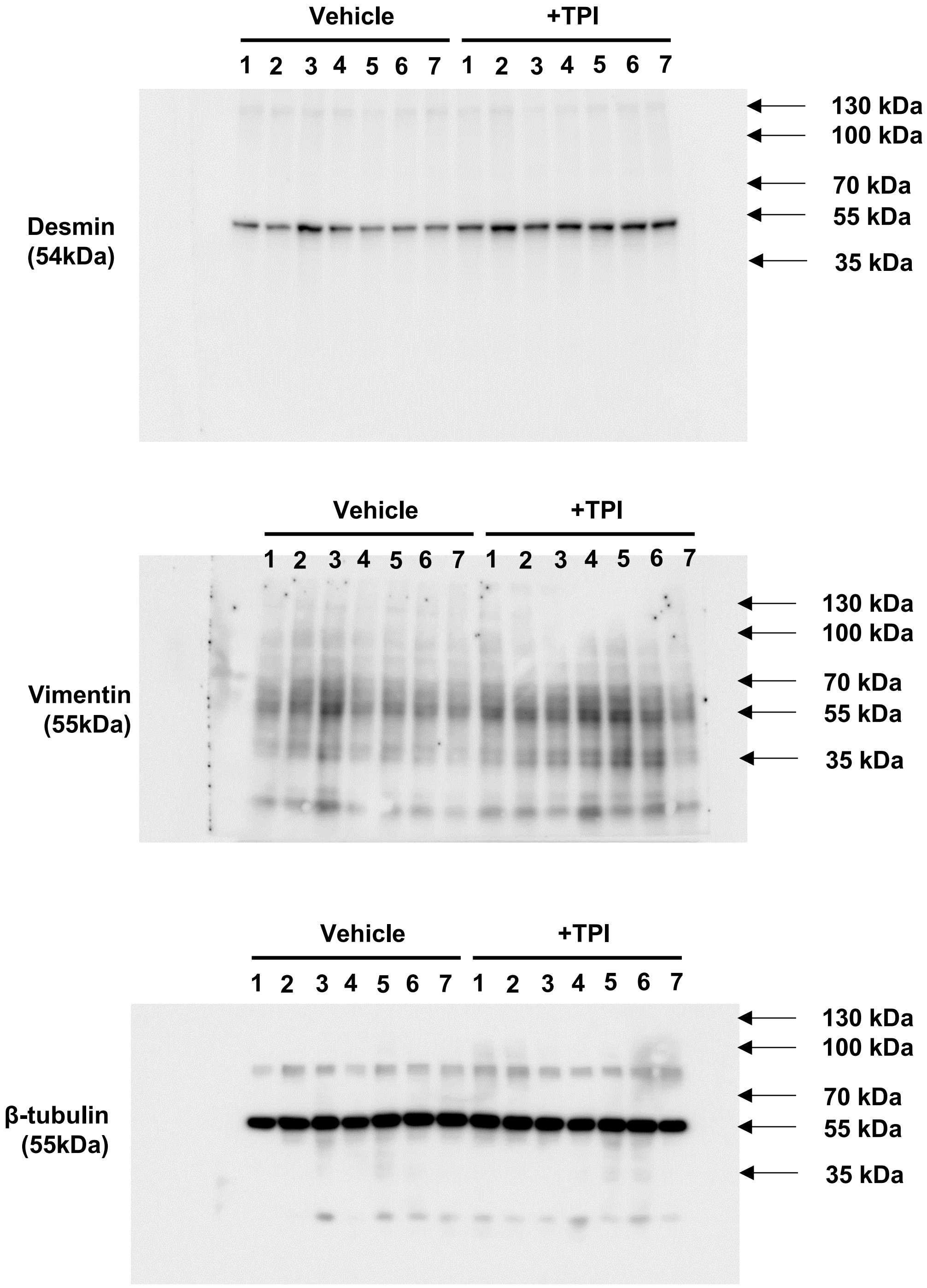

Supplement: Unedited blot and gel images [file jci-134-180310-s007.pdf]
